# Learning the syntax of plant assemblages

**DOI:** 10.1038/s41477-025-02105-7

**Published:** 2025-10-13

**Authors:** César Leblanc, Pierre Bonnet, Maximilien Servajean, Wilfried Thuiller, Milan Chytrý, Svetlana Aćić, Olivier Argagnon, Idoia Biurrun, Gianmaria Bonari, Helge Bruelheide, Juan Antonio Campos, Andraž Čarni, Renata Ćušterevska, Michele De Sanctis, Jürgen Dengler, Tetiana Dziuba, Emmanuel Garbolino, Ute Jandt, Florian Jansen, Jonathan Lenoir, Jesper Erenskjold Moeslund, Aaron Pérez-Haase, Remigiusz Pielech, Jozef Sibik, Zvjezdana Stančić, Domas Uogintas, Thomas Wohlgemuth, Alexis Joly

**Affiliations:** 1 Inria, LIRMM, Université de Montpellier, CNRS, Montpellier, France; 2 AMAP, Université de Montpellier, CIRAD, CNRS, INRAE, IRD, Montpellier, France; 3 LIRMM, AMIS, Université de Montpellier Paul Valéry, CNRS, Montpellier, France; 4 Université Grenoble Alpes, Université Savoie Mont Blanc, CNRS, LECA, Grenoble, France; 5 Department of Botany and Zoology, Faculty of Science, Masaryk University, Brno, Czech Republic; 6 Department of Botany, Faculty of Agriculture, University of Belgrade, Belgrade-Zemun, Serbia; 7 Conservatoire Botanique National Méditerranéen, Hyères, France; 8 Department of Plant Biology and Ecology, University of the Basque Country UPV/EHU, Bilbao, Spain; 9 Department of Life Sciences, University of Siena, Siena, Italy; 10 Institute of Biology/Geobotany and Botanical Garden, Martin Luther University Halle-Wittenberg, Halle, Germany; 11 German Centre for Integrative Biodiversity Research Halle-Jena-Leipzig, Leipzig, Germany; 12 Research Centre of the Slovenian Academy of Sciences and Arts, Jovan Hadži Institute of Biology, Ljubljana, Slovenia; 13 School for Viticulture and Enology, University of Nova Gorica, Nova Gorica, Slovenia; 14 Institute of Biology, Faculty of Natural Sciences and Mathematics, Ss. Cyril and Methodius University, Skopje, Republic of North Macedonia; 15 Department of Environmental Biology, Sapienza University of Rome, Rome, Italy; 16 Vegetation Ecology Research Group, Institute of Natural Resource Sciences, Zurich University of Applied Sciences, Wädenswil, Switzerland; 17 Bayreuth Center of Ecology and Environmental Research, University of Bayreuth, Bayreuth, Germany; 18 Department of Geobotany and Ecology, M.G. Kholodny Institute of Botany, National Academy of Sciences of Ukraine, Kyiv, Ukraine; 19 Mines Paris PSL-ISIGE, Fontainebleau, France; 20 Faculty of Agricultural and Environmental Sciences, University of Rostock, Rostock, Germany; 21 UMR CNRS 7058 ‘Ecologie et Dynamique des Systèmes Anthropisés’, Université de Picardie Jules Verne, Amiens, France; 22 Department of Ecoscience, Aarhus University, Aarhus C, Denmark; 23 Department of Evolutionary Biology, Ecology, and Environmental Sciences, University of Barcelona, Barcelona, Spain; 24 Biodiversity Research Institute, University of Barcelona, Barcelona, Spain; 25 Institute of Botany, Faculty of Biology, Jagiellonian University in Kraków, Kraków, Poland; 26 Plant Science and Biodiversity Center, Slovak Academy of Sciences, Bratislava, Slovak Republic; 27 Faculty of Geotechnical Engineering, University of Zagreb, Varaždin, Croatia; 28 State Scientific Research Institute Nature Research Centre, Vilnius, Lithuania; 29 Swiss Federal Institute for Forest, Snow and Landscape Research WSL, Birmensdorf, Switzerland; 30 Present Address: Institut National de Recherche en Sciences et Technologies du Numérique, Le Chesnay-Rocquencourt, Île-de-France, France

**Keywords:** Conservation biology, Plant ecology, Biogeography, Ecological modelling, Ecosystem ecology

## Abstract

To address the urgent biodiversity crisis, it is crucial to understand the nature of plant assemblages. The distribution of plant species is shaped not only by their broad environmental requirements but also by micro-environmental conditions, dispersal limitations, and direct and indirect species interactions. While predicting species composition and habitat type is essential for conservation and restoration purposes, it remains challenging. In this study, we propose an approach inspired by advances in large language models to learn the ‘syntax’ of abundance-ordered plant species sequences in communities. Our method, which captures latent associations between species across diverse ecosystems, can be fine-tuned for diverse tasks. In particular, we show that our methodology is able to outperform other approaches to (1) predict species that might occur in an assemblage given the other listed species, despite being originally missing in the species list (16.53% higher accuracy in retrieving a plant species removed from an assemblage than co-occurrence matrices and 6.56% higher than neural networks), and (2) classify habitat types from species assemblages (5.54% higher accuracy in assigning a habitat type to an assemblage than expert system classifiers and 1.14% higher than tabular deep learning). The proposed application has a vocabulary that covers over 10,000 plant species from Europe and adjacent countries and provides a powerful methodology for improving biodiversity mapping, restoration and conservation biology. As ecologists begin to explore the use of artificial intelligence, such approaches open opportunities for rethinking how we model, monitor and understand nature.

## Abstract (web summary)

Pl@ntBERT is a language-based AI model that learned the ‘syntax’ of plant assemblages, predicting likely species and inferring habitats by modelling biotic relationships.

## Main

Understanding vegetation patterns and plant assemblages is central to ecology, as co-occurring species ultimately determine the structure and function of ecosystems^1^. Plant species rarely exist in isolation^2^; instead, they form complex assemblages influenced by biotic and abiotic conditions^3^. These assemblages represent the emergent properties of ecosystems, where each species contributes to and is influenced by the broader assemblage^4^. Identifying and analysing these intricate patterns is crucial for understanding the underlying mechanisms governing biodiversity and ecosystem stability and dynamics^5^. Despite progress, unravelling these patterns remains challenging, given the high dimensionality and complexity of community assembly^6^. In this study, we attempt to decode the ‘syntax’ of plant community structure, aiming to provide insights on the composition of vegetation across diverse ecosystems. In this context, ‘syntax’ refers to the implicit rules and patterns that govern how plant species co-occur and interact to form structured assemblages, similar to how syntax in language defines the arrangement of words to create meaningful sentences. Just as language syntax reveals relationships between words on the basis of their positions and roles, the syntax of plant assemblages represents the hidden shared environmental preferences, direct and indirect interactions, and organization underlying species assemblages (that is, just as the ordering of words in a sentence matters, the ranking of species names in a community matters as well). We focus particularly on how this approach can be used to improve habitat type identification, offering insights that could enhance ecological classification and conservation efforts.

The analysis of species communities is often done by leveraging presence–absence matrices of species co-occurrences^7^, which record how many times two different species were observed together in the same vegetation plot. This traditional approach allows for global analyses of co-occurrence patterns in vegetation plots found in a dataset, making it suitable for detecting broad patterns, such as clusters of species with a high tendency of co-occurrence^8^. However, this method is often biased towards common species^9^, as they have higher occurrence frequencies across vegetation plots, leading to inflated co-occurrence estimates. This can obscure the detection of rare or specialized species interactions^10^, which may play critical ecological roles but are underrepresented in presence–absence matrices.

To address this limitation, alternative approaches such as fidelity indices^11^ quantify species’ specificity to particular habitat types rather than relying solely on their co-occurrence frequencies, making these approaches particularly useful for distinguishing diagnostic species from widely distributed ones. While such methods might offer an improvement over raw co-occurrence counts, they remain constrained by predefined habitat classifications and do not fully capture the hierarchical and context-dependent nature of species associations. In addition, most co-occurrence matrices only account for species presence or absence in the assemblage, but the relative abundance of species within plant assemblages, which is often important for habitat and vegetation classification^12^, is not taken into account. Statistical interdependencies, which reflect biotic interactions, often exhibit asymmetric, transitive and hierarchical patterns^13^ that are beyond the scope of classical co-occurrence approaches but can be captured by more recent and sophisticated AI-based abundance-order language models. These models use a transformer-type deep learning architecture based on self-attention mechanisms (which allow the model to weight the importance of each species in relation to all others in a given assemblage, much like how one might focus on key words in a sentence to understand its meaning). This allows such a model to account for bidirectional dependencies in a statistical sense (that is, in the extent to which the presence or abundance of one or several species helps predict others), not necessarily reflecting ecological causality. These patterns include asymmetries (that is, if species A statistically influences species B but species B does not necessarily statistically influence species A), indirect relationships such as transitivities (that is, if species A statistically influences species B and species B statistically influences species C, then species A statistically influences species C) and hierarchical patterns in the assemblage (for example, abundant species that tend to co-occur with other less abundant species).

A concrete application of the model evaluated in our study is the classification of European habitat types based on ordered species assemblages. Europe hosts a rich diversity of vascular plant species, contributing to a great number of unique habitats^14^ shaped by both biotic and abiotic factors and protected by the European Habitats Directive. However, this biodiversity faces many threats, including the effects of various kinds of agricultural activities (for example, intensification for more productive farming and abandonment of traditional land use) and modifications of natural systems (for example, dredging and sea defence works), among others^15^. All habitats protected by the Habitat Directive are listed in Annex I of this directive^16^, and with the new European Union restoration law, a large proportion of these habitats have to be in a favourable state in the near future^17^. A major challenge is that in many European Union countries, only a fraction of these habitats have been mapped, making it difficult to monitor their development and condition. Moreover, even when mapped, their ecological quality often remains unknown, further complicating conservation and management efforts. Here we try to patch this major knowledge gap.

For the purpose of this study, habitats were defined as terrestrial, freshwater or marine areas characterized by geographic, abiotic and biotic features^18^. We leveraged the European Nature Information System (EUNIS)^19^ maintained by the European Environment Agency. This hierarchical classification system covers all types of habitats and contains at least five levels of complexity. We focused our analysis on the first three levels: broad habitat groups (level one), habitat groups (level two) and habitat types (level three). Specifically, our experiments concentrated on predicting habitat types that are within eight broad habitat groups. It is important to note that habitat types, such as those defined by the EUNIS typology, are human-constructed categories that impose structure on a continuum of vegetation patterns.

Habitat distribution modelling typically involves linking information on plant species composition (such as a full list of vascular plant species with estimates of cover abundance) and environmental covariates (such as whether a community is located on a coastal dune^20^ or within a specific terrestrial ecoregion^21^) to habitat type occurrences. This approach helps identify the habitat type of vegetation plots. There are two basic types of methodologies used for vegetation classification based on species composition^22^: expert systems^23^ and machine learning^24^. The former leverage explicitly defined logical rules and emulate the process of expert classification done by humans, whereas the latter are tools for induction of the independent knowledge base.

Expert systems, even though they are still the most used tools to assign plots to vegetation types, do not consistently align with the basic requirements for vegetation classification^25^:

- They tend to overfit by learning the detail in the training data too well. Thus, minor changes in a vegetation plot (for example, a small difference in the cover of an individual species) can considerably alter the result of the classification procedure, making those expert systems not robust.
- Some of them involve sets of external criteria (for example, environmental or geographical attributes of vegetation plots in addition to species composition) to classify some vegetation types, making those expert systems not simple.
- They are often based on one specific nomenclatural and taxonomic dataset, but using vegetation plots from different origins might result in different names for the same entity or identical names for different entities (depending on the taxonomic concepts and determination literature used in a particular region or period), making those expert systems not consistent.

Modern deep learning techniques have great potential for modelling habitat distributions^26^. In particular, experiments with feedforward neural networks have shown that they have the ability to capture complex information about the plant species composition of vegetation plots to classify plant communities^27^. One limitation of such models, however, is that their architecture induces an intrinsic inductive bias in the sense that they process each plant species as if it is equally different from all the others^28^. Thus, they cannot accurately model complex relationships between plant species. They are therefore not really suitable for modelling ecological systems and identifying habitat types where the interdependencies between plant species are complex^29^. Classical approaches offer interpretable and mathematically grounded methods for ecological modelling^30^. However, they may lack the capacity to learn latent patterns from high-dimensional data, such as subtle co-occurrence relationships between plant species, hierarchical community structures or environmental gradients that shape species assemblages.

In contrast, transformers^31^, a different kind of deep learning model, go beyond local processing and exploit global attention mechanisms for increased performance. Although transformers have been leveraged in various fields of biology (for example, the extraction of morphological traits^32^ or the prediction of protein structures^33^), their use in vegetation classification is still largely unexplored. Such models should allow the segmenting of habitats in a much more efficient manner than current methods. In particular, large language models (LLMs) have not yet been embraced by the global community of ecologists despite their ability to find patterns and correlations in noisy biological data^34^.

**Fig. 1:**
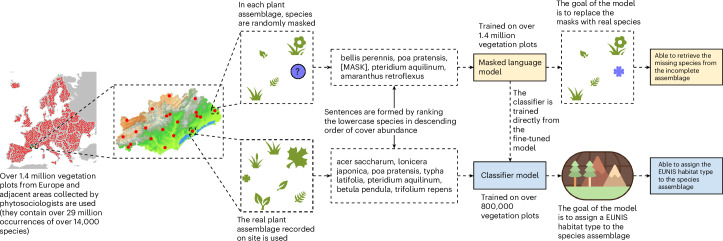
The proposed approach leverages LLMs to capture the latent dependencies between plant species in diverse ecosystems. By training on over 1.4 million vegetation plots, 29 million species occurrences and 14,000 species from Europe and adjacent regions, the model learns the ‘syntax’ of sentences formed by abundance-ordered plant species sequences, allowing it to predict missing (that is, [MASK]) taxa in sequences of species. The resulting foundation model can be further fine-tuned to assign EUNIS habitat types to vegetation plots, outperforming traditional methods on both tasks.

The goal of this work is to enhance the understanding of species assemblages and facilitate habitat identification in Europe through the use of LLMs (Fig. 1). To achieve this goal, we introduce a computational pipeline centred on Pl@ntBERT^35^, a model based on BERT^36^ (that is, Bidirectional Encoder Representations from Transformers, a deep learning model originally designed for natural language understanding). This means that without any further adaptation (that is, fine-tuning), Pl@ntBERT would be pretrained only in a self-supervised manner on very large volumes of common text data unrelated to vegetation (that is, BookCorpus and English Wikipedia) and would be a Swiss army knife solution (that is, this model would work for the most common language tasks, such as sentiment analysis or named entity recognition, as long as they did not require a deep knowledge of the domain). However, to make it ecologically meaningful, we pretrain it (that is, we make the model learn the general structure in the data) on an in-domain dataset named the European Vegetation Archive (EVA)^37^, an integrated database of European vegetation plots. This adaptation allows Pl@ntBERT to develop a statistical representation of the vegetation assemblages, capturing implicit relationships between species that commonly co-occur, and boost the performance of the downstream task (for example, keeping the learned features but replacing the final layer improves habitat type identification).

The next step is to train the model for a supervised classification task: assigning habitat types to species assemblages. We use the EUNIS classification system, a widely used European framework that organizes vegetation into hierarchical habitat types based primarily on dominant species composition, ecological structure and environmental conditions. The EUNIS typology provides a standardized way to classify and compare habitats across Europe, making it a key reference for conservation and land management. However, as EUNIS is a human-constructed typology, it has to be noted that sometimes, the habitat type labels that were assigned to vegetation plots by the vegetation scientists that collected the data may be ambiguous or uncertain. Unlike traditional expert systems, which rely on manually defined classification rules, or classical machine learning approaches, which process species independently without considering their ecological interdependencies, Pl@ntBERT learns to infer habitat types by recognizing patterns in species composition and their statistical relationships. This approach enhances classification accuracy, mitigates inconsistencies in taxonomic nomenclature (by accommodating variation in species names such as synonyms) and provides a scalable solution for habitat identification, including for habitats under threat of collapse.

## Results

### The syntax of species assemblages

Understanding the structure of species assemblages requires capturing both direct and indirect relationships between co-occurring species. To measure Pl@ntBERT’s ability to capture these complex relationships from abundance-ordered species communities, we evaluated it on a so-called masking or fill-mask task (that is, a species is removed from the assemblage, and the accuracy of the model in recovering the right species is measured). This approach is conceptually related to the notion of dark diversity^38^, as it aims to identify missing species that, on the basis of the ecological context, are expected to be present but are absent in a given assemblage. For this evaluation, we tested different versions of Pl@ntBERT, which vary in how they tokenize species names (refer to Methods for more details about these different versions). Naturally, the models using a tokenizer where species names are split into two tokens (one for the genus name and one for the species epithet) tend to perform better in the masked token prediction task. This result is expected, since each mask hides only half of the species name rather than the entire binomial name. As a result, it is easier for these models to figure out what the other half of the binomial name is (for example, ‘*Thinopyrum junceum*, [MASK] *marina*, *Pancratium maritimum*’). In contrast, the models using a tokenizer where species names are considered as one single token have to choose between over 14,000 different species to replace the mask, which completely hides a species name (for example, ‘*Thinopyrum junceum*, [MASK], *Pancratium maritimum*’), making the task harder.

**Fig. 2:**
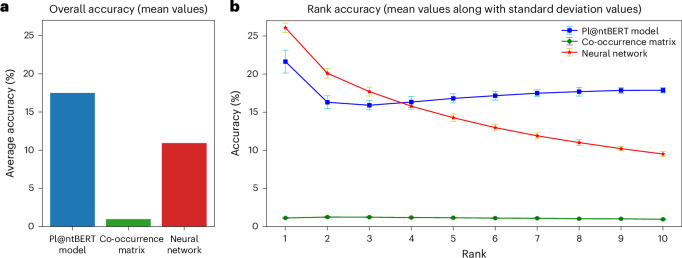
Overall masking accuracy (micro-averaged over the ten cross-validation folds) of the three methods and breakdown of the rank accuracy. **a**, Overall accuracy (mean values). **b**, Rank accuracy (mean values along with standard deviations). Only the labelled vegetation plots for which over ten species were recorded were kept in the test set. For each remaining vegetation plot (*n* = 705,479), the ten most abundant species were masked one by one, and the accuracy corresponding to each species rank was computed.

To assess how well Pl@ntBERT captures species relationships beyond simple co-occurrences, we conducted a comparative evaluation against two alternative approaches: (1) a naive Bayes model^39^ using only the species co-occurrence matrix and (2) a classical deep learning model^40^ based on a feedforward neural network (Fig. 2). This comparison allowed us to determine whether Pl@ntBERT’s ability to encode species assemblages translates into improved predictive power when identifying missing species in vegetation plots. Pl@ntBERT clearly outperforms the co-occurrence matrix at every rank—that is, at every position that species can occupy in the vegetation plot when they are sorted by cover abundance (Fig. 2b). Moreover, the co-occurrence matrix tends to perform worse when the species is less abundant. The neural network is very good for the most dominant species, even outperforming the Pl@ntBERT model on the first ranks. However, when the species become less abundant, it quickly loses its predictive power. In contrast, the Pl@ntBERT model tends to perform better for scarce species than for abundant species. Indeed, the accuracy of its predictions drops sharply when the first ranked species (most abundant) are masked (from around 22% to around 16% for species ranked second to third) but then slowly increases for species ranked after (and stabilizes around 18% for species ranked tenth). This indicates that, as the first species is the one contributing the most to the assemblage structure and identity, it is more likely for our model to find it if it has complete knowledge of the assemblages (that is, all other species), especially the second and third species. Moreover, it shows that the presence of abundant species is essential but not sufficient to determine the assemblage. However, the assemblage of the first three species (and also the assemblage of only the second and third species) is often sufficient to determine the ecosystem. This emphasizes the critical role that species abundance plays in accurately predicting missing species in an assemblage. As it is often the rarer and less abundant species that are missing from vegetation-plot records, this experiment highlights the importance of using models like Pl@ntBERT to capture nuanced relationships between species. See appendix 36 in the Supplementary Information for a more in-depth overview of each method’s result.

The task of finding missing species from highly diverse, incomplete plant assemblages benefits notably from the ability to capture complex relationships, leverage extensive textual data for contextual understanding and learn rich, abstract data representations. A comparison between the results obtained by the Pl@ntBERT model, the co-occurrence matrix and the neural network (Supplementary Fig. 12) shows that the LLM clearly outperforms the other two approaches in this regard. LLMs provide a holistic view that aids in recognizing patterns and improving species identification. The co-occurrence matrix relies on simple frequency counts of species pairs appearing together in the training dataset^41^, and the neural network relies on one-hot encoded assemblages of co-occurring species^42^, which lack the contextual understanding necessary to accurately predict the masked tokens in a complex and domain-specific dataset such as plant species names. Whatever the broad habitat groups (for example, vegetated man-made habitats, wetlands, forests and other wooded land), Pl@ntBERT consistently outperforms the co-occurrence matrix by a factor of more than ten and, except for littoral biogenic habitats and coastal habitats, the neural network by a factor of almost two (overall accuracy of 17.49% for the Pl@ntBERT model, 0.96% for the co-occurrence matrix and 10.93% for the neural network; Fig. 2a).

Furthermore, we show that Pl@ntBERT is able to perform better than both the co-occurrence matrix and the neural network when detecting species patterns (appendix 29 in the Supplementary Information). In scenarios where three species A, B and C occur together more than 100 times in a vegetation plot but where species A and species C never occur together without species B, Pl@ntBERT is often able to predict that the species B is required for the presence of the other two species, unlike the other methods. In contrast, the co-occurrence matrix and the neural network repeatedly predict common species (for example, *Dactylis glomerata*, which is the most frequent species in the dataset, or *Phragmites australis*), even in cases where they are not plausible candidates, showing a tendency to favour species that appear many times in the dataset rather than recognizing specific ecological patterns. Pl@ntBERT’s success demonstrates its capacity to learn the complex syntax of plant assemblages and correctly identify species occurrence relationships, even in complicated ecological contexts. Practically, Pl@ntBERT can support vegetation surveys by suggesting species that are probably present but unrecorded. After conducting an initial survey and recording a set of species, one can append [MASK] tokens sequentially to the end of the observed species list. At each iteration, the model outputs probabilities over all tokens, both species tokens and special tokens—including the [SEP] token, which indicates the end of the sentence (that is, the end of the list). When [SEP] has the highest probability, it indicates that the model considers the assemblage complete, hence providing a natural stopping criterion without prior knowledge of total species richness. This capability can also help flag potential omissions or inconsistencies in species lists. By offering context-aware predictions, the model can act as a quality-control tool that complements field observations and contributes to more complete and reliable habitat assessments. Indeed, observer errors (for example, overlooking errors and misidentification errors) may result in species richness being artificially underestimated^43^. This fill-mask task can thus support rapid floristic assessments, where only dominant or easily identified species are observed, by predicting likely missing species.

The task of finding a missing species in an assemblage is a complex problem, as the hypothesis space is large. Indeed, when asked to replace a [MASK] token in a sentence describing a vegetation plot, the model Pl@ntBERT must select from over 14,000 different vascular plant species. However, the perplexity of the base model indicates that it mostly hesitates between around 12 species when it has to replace the mask. More importantly, an experiment shown in Supplementary Fig. 15 indicates the following:

- When the Pl@ntBERT model (the large-species version) does not replace the [MASK] token with the correct species, it actually outputs a species coming from the same vegetation class^44^ (that is, a species belonging to the same broad unit in a hierarchical classification system that groups plant communities on the basis of shared floristic composition, ecological characteristics and biogeography) over 39% of the time. For comparison, a random approach (that is, predicting a random species to replace the [MASK] token) would result in a species coming from the same vegetation class around 3.5% of the time. Pl@ntBERT thus provides a substantial improvement over chance, especially considering there are over 100 vegetation classes in the classification system, many of which share ecologically similar species that may co-occur across different vegetation classes.
- When the Pl@ntBERT model (the large-species version) does not replace the [MASK] token with the correct species, it actually outputs a species that is characteristic of the habitat type (level 3) of the vegetation plot 49% of the time, of the habitat group (level 2) 66% of the time and of the broad habitat group (level 1) 76% of the time. For comparison, a random approach would result in a species being characteristic of the habitat type of the vegetation plot 0.3% of the time, of the habitat group 2.3% of the time and of the broad habitat group 7.0% of the time. Again, Pl@ntBERT provides a substantial improvement over chance, especially considering there are hundreds of habitat types in the classification system, many of which share ecologically similar species that may co-occur across different habitat types.

**Fig. 3:**
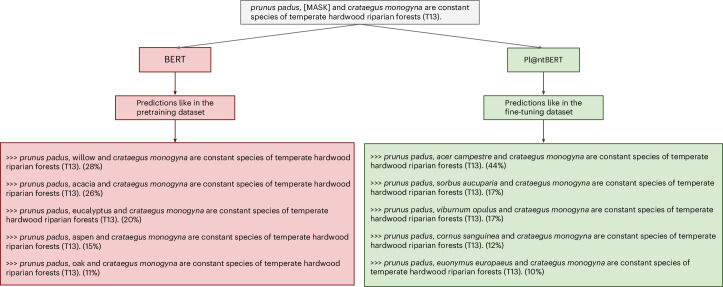
Comparison of the top five predictions for the BERT (large-uncased version) and Pl@ntBERT (large-species version trained on folds 1–9) models for our sample text of ‘*prunus padus*, [MASK] and *crataegus monogyna* are constant species of temperate hardwood riparian forests (T13)’. The percentages next to each predicted species represent the probabilities assigned by the models for replacing the [MASK] token, normalized so that the top five predictions sum to 100%. On the one hand, the candidates from BERT are all trees, which shows that the model ‘understood’ we are in a forest. However, all of them are common plant names (and not scientific names of taxa) and, except for the oak, which is the last candidate, are not found within the T13 habitat type. On the other hand, the candidates from Pl@ntBERT are all scientific names of constant species from the required habitat type.

A comparison of the vocabularies of different models can be found in appendix 18 in the Supplementary Information. For example, *verticillatoinundata*, a species epithet, is divided into eight pieces ([ve, ##rti, ##ci, ##lla, ##to, ##in, ##unda, ##ta]) by BERT and into seven pieces ([ver, ##tic, ##illa, ##to, ##in, ##und, ##ata]) by SciBERT^45^ (that is, a BERT model trained on scientific text). In contrast, this term appears in the in-domain vocabulary of Pl@ntBERT, as well as around 10,000 other genus names and species epithets. Species names are specific, meaningful biological entities. Splitting them into multiple smaller components (referred to as subwords in machine learning terminology) blocks the model’s ability to recognize these tokens as representing a unified biological entity. Instead of treating the entire species name as a single, coherent unit, the model sees it as a collection of unrelated fragments, which reduces its ability to capture biological relationships. An example of the benefits of domain adaptation is shown in Fig. 3. It shows that Pl@ntBERT (that is, a fine-tuned BERT), compared with a vanilla BERT (that is, the standard, pretrained BERT model not specialized for plant-related data), really ‘understands’ plant species compositions. A visualization of the attention in Pl@ntBERT can be found in Supplementary Fig. 8. This makes the model more accessible and shows at multiple scales which species in a vegetation plot most influence the predictions.

### Identifying habitat types

To optimize the hyperparameters (that is, learning rate and batch size) and identify the set of parameters yielding the most accurate model, we first fine-tuned all versions of Pl@ntBERT using the first fold as a test set and the remaining nine folds as a training set. All results obtained during this fine-tuning process can be found in Supplementary Table 4. Table 1 gives an overview of the results obtained in the text classification task, and Supplementary Fig. 5 provides more details. Among all tested models, Pl@ntBERT-large-species appears as the clear winner when it comes to identifying habitat types. It outperforms all other models, whether it is on top-one accuracy (that is, the first candidate output by the model is the real habitat type, or level 3 habitat), top-three accuracy (that is, among the three first candidates output by the model is the real habitat type, or level 3 habitat), group accuracy (that is, the first candidate output by the model belongs to the real habitat group, or level 2 habitat) or broad accuracy (that is, the first candidate output by the model belongs to the real broad habitat group, or level 1 habitat). It also outperforms models that, in addition to species composition, use the abiotic environment and geographic location as classification criteria. The different versions of the expert system EUNIS-ESy and the different models of hdm-framework, as statistical and general-purpose machine learning approaches, are not capable of matching domain-adapted models such as Pl@ntBERT for specialized tasks in vegetation classification.

**Table 1:** Comparison of Pl@ntBERT, the expert system EUNIS-ESy and the deep learning models from hdm-framework (with the settings recommended by the authors) for habitat type classification

Framework	Model	Fine-tuning			
		Accuracy (%)	Top-three accuracy (%)	Group accuracy (%)	Broad accuracy (%)
Predictors: species composition, abiotic environment and geographic location					
EUNIS-ESy	v.2020-06-08	82.68	–	84.34	90.72
	v.2021-06-01	86.44	–	88.26	94.64
hdm-framework	MLP^72^	90.84	98.90	93.94	95.79
	RFC^73^	80.37	95.73	87.85	92.13
	XGB^74^	88.81	98.95	93.00	95.69
	TNC^75^	81.50	92.13	87.11	90.70
	FTT^76^	88.84	97.28	92.65	94.92
Predictors: species composition					
hdm-framework	MLP	90.00	98.73	93.36	95.27
	RFC	80.34	95.66	87.82	92.00
	XGB	88.11	98.75	92.60	95.29
	TNC	80.64	91.73	86.40	89.98
	FTT	87.92	97.06	92.08	94.40
Pl@ntBERT (ours)	Large-species	**91.98**	**99.10**	**94.79**	**96.42**

All models were evaluated using the same ten cross-validation folds. Predictions were made at level 3 of the EUNIS hierarchy, with group (level 2) and broad (level 1) accuracies derived from the habitat types. EUNIS-ESy and hdm-framework^77^ used additional location covariates (for example, country, ecoregion and elevation), while Pl@ntBERT used species composition only (hdm-framework was also evaluated without location covariates). EUNIS-ESy uses the exact cover abundance of all species instead of their relative ranks. An en dash indicates that the cell is not applicable or relevant for the corresponding model. Bold indicates the best-performing model per metric. See Supplementary Text 6 for metric definitions.

**Fig. 4:**
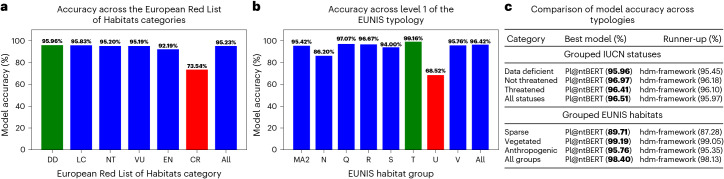
Accuracy obtained by the Pl@ntBERT-large-species model on different typologies (results averaged over the ten cross-validation folds). **a**, Accuracy results (in %, with the best accuracy in green and the worst accuracy in red) of Pl@ntBERT across the European Red List of Habitats categories (DD, data deficient; LC, least concern; NT, near threatened; VU, vulnerable; EN, endangered; CR, critical endangered). **b**, Accuracy results (in %, with the best accuracy in green and the worst accuracy in red) of Pl@ntBERT across level 1 (that is, broad habitat groups) of the EUNIS typology (MA2, marine; N, coastal; Q, wetlands; R, grasslands; S, heathlands; T, forests; U, inland; V, man-made). **c**, Comparison of the accuracy (in %, best accuracy in bold) of the models across grouped European Red List of Habitats statuses (data deficient, {DD}; not threatened, {LC + NT}; threatened, {VU + EN + CR}) and EUNIS broad habitat groups (sparse, {MA2 + U}; vegetated, {R + N + Q + S + T}; anthropogenic, {V}).

Pl@ntBERT (the large-species version) achieves an accuracy of 92% when asked to classify a vegetation plot into one of the 227 habitat types present in the dataset. More details on how some habitat groups are sometimes confused with other habitat groups can be found in Supplementary Fig. 13. As shown in Fig. 4, when assessing the risk of habitat collapse (after converting the predictions from EUNIS habitat types to European Red List of Habitats categories), Pl@ntBERT achieves an overall micro-accuracy of 96.5%. Furthermore, our transformer-based method outperforms all other approaches (Table 4c) and shows very strong accuracy when identifying individual conservation statuses (Fig. 4a) and broad habitat groups (Fig. 4b). As a result, Pl@ntBERT can be seen as a powerful tool to inform and catalyse action for biodiversity conservation and policy change. More details about the distribution of the European Red List of Habitats categories across the dataset can be found in appendix 27 in the Supplementary Information. We used this model to map all the unlabelled vegetation plots from the dataset, and we compare the output with the map of all labelled vegetation plots from the dataset in appendix 33 in the Supplementary Information (with a further breakdown on each individual broad habitat group from the fill-mask dataset in appendix 34 in the Supplementary Information).

Some other experiments (shown in Supplementary Fig. 17) demonstrated that the most important species for identifying the habitat type of a vegetation plot are the first ones in the cover-abundance rank. Indeed, over all the vegetation plots of the dataset containing ten species or more, Pl@ntBERT-large-species achieves an accuracy of 92.2%. When the first species (that is, the most abundant) of each vegetation plot is removed, the accuracy drops by 35 percentage points to 57.2%. When the last species (that is, the least abundant) of each vegetation plot is removed, the accuracy almost stays the same and drops by only 0.43 percentage points (91.7%). When a random species from each vegetation plot is removed, the accuracy decreases by 3.0 percentage points to 89.2%. This discrepancy probably arises because dominant species shape the ecological structure of habitats. These results highlight the strong influence of dominant species in habitat type identification, while rare species contribute minimally to the model’s predictive performance. This could allow less well-trained botanists who know only common and/or abundant species to conduct field surveys and still identify the habitat of the area while speeding up data collection.

### Open science

To facilitate the reproducibility of our study and the reuse of codes and models, we develop, share and maintain a generic, free and open-source deep learning framework facilitating the training and evaluation of predictive models of habitats from in situ observation data and inference on new and unseen vegetation-plot records. The framework, coded in the programming language Python and powered by the parallel computing platform CUDA for accelerated training and inference, is accessible to various user profiles (including non-deep-learning experts who want to easily identify European habitat types) at https://github.com/cesar-leblanc/plantbert. A user guide on how to install the framework and run the basic tasks (that is, data curation, fill-mask training, text classification training and inference) can be found in Supplementary Text 20, and some examples of how the model works can be found Supplementary Text 23. If the user has only a few vegetation plots from which they want to find potentially missing species or identify the habitat type, a quicker way to test the framework is to visit the tool available at https://huggingface.co/spaces/CesarLeblanc/plantbert_space. A demo can be found in Supplementary Fig. 19.

## Discussion

The Pl@ntBERT model has been created to offer insights into how vegetation patterns can be encoded and classified, contributing to advancements in plant ecology and conservation biology. It introduces an innovative approach by leveraging natural language processing techniques on top of abundance-ordered species lists from specific sites aimed at capturing complex species relationships such as transitive or sequential dependencies. As a result, it can model the species composition of hundreds of terrestrial, freshwater and marine habitat types that contain plants, including most of the threatened, vulnerable and endangered ecosystems found across Europe and adjacent areas. In addition, this approach can be expanded worldwide—for example, by applying it to the global vegetation-plot database sPlot^46^.

The model has been primarily designed to predict missing species in an assemblage (which can also be used for predicting species pools of plant assemblages^47^)—for example, in incomplete monitoring projects^48^, leveraging masked language modelling to infer statistically probable species compositions, hence enhancing species completeness and improving vegetation surveys. This capability is especially relevant in cases where survey data may be incomplete or where one or more species could be overlooked due to sampling limitations or observer bias. By simulating the expected species pool, Pl@ntBERT offers a means to improve the ecological relevance of data used for habitat assessments, management and reporting. This predictive function can support the identification of indicator species and enhance the detection of key ecological patterns that may be otherwise underrepresented. However, although Pl@ntBERT can predict missing species in incomplete assemblages, caution is needed when interpreting these predictions. In some cases, a species’ absence from a vegetation plot might be due to observer bias or sampling limitations, in which case its predicted presence could be justified. But some absent species belong to dark diversity (that is, species expected to occur on the basis of ecological conditions but that are genuinely missing due to dispersal limitations, competition or other constraints). In such cases, attempting to ‘correct’ field surveys by adding model-predicted species risks misrepresenting reality and creating fictional plots, which could introduce more error than it solves. From an ethical standpoint, modifying field data in this way might also be controversial, as it could lead to unintended biases in conservation and management decisions. Incomplete data are an inherent part of ecological research, and rather than filling gaps artificially, it might sometimes be preferable to acknowledge and work with these uncertainties.

The second key application of Pl@ntBERT is its capacity to classify plant species records into EUNIS habitat types. This ability addresses an essential need in habitat identification and conservation planning, where the ability to classify survey data is foundational for monitoring biodiversity and guiding restoration efforts. Traditional methods have largely relied on manual expertise or rigid algorithms that cannot capture the complex patterns and overlook associations that occur in large ecological datasets. By leveraging transformer-based architectures and fine-tuning them with domain-specific botanical datasets, Pl@ntBERT offers a more refined and accurate approach. It is also worth noting that some vegetation plots in the EVA database may represent transitional or ecotonal habitats that do not fit neatly into a single EUNIS type. Such cases introduce ambiguity in classification and may contribute to an underestimation of Pl@ntBERT’s true accuracy, as the model, even though capable of assigning multiple habitat types to a vegetation plot, is evaluated on the task of assigning only one, which might be ecologically reasonable but could differ from the labelled category (in this case, considering the top-three accuracy might be wiser). It is also important to consider potential regional biases due to uneven plot densities in EVA. Some habitat types may be disproportionately represented in well-surveyed regions, leading the model to learn patterns that reflect data availability rather than true ecological distributions. This could result in higher accuracy for frequently sampled habitats and reduced performance for underrepresented ones.

By learning the context to translate plant species into a modelled ecological process within an ecosystem, Pl@ntBERT is able to improve vegetation models for identifying habitat types. This domain adaptation helps the model automatically understand that some species occur only in very specific assemblages, while others can tolerate and thrive in a wide range of ecosystems. Predictions are therefore influenced not only by the actual occurrence of a given species but also by the relative probability of the presence of this species. However, some habitat types, such as those listed in Annex I, are not defined solely by vegetation but rather by geomorphological or geolocational parameters (for example, springs, cliffs and dune slacks). These features are unlikely to be predictable by Pl@ntBERT, as they do not necessarily correlate with species composition alone. Similarly, certain species-poor EUNIS habitat types present challenges for classification since their low species richness limits the available signal for distinguishing between communities. Moreover, in few cases, it is impossible to distinguish some habitat types by plant species composition and relative abundance alone, because their species composition can be the same even if they occur in different regions of the world. This is one of the main reasons why attribute data (for example, coordinates, country and elevation) were incorporated in expert-based systems such as EUNIS-ESy, rather than relying purely on species presence.

The relative positions of the species within a vegetation plot (that is, their abundance compared to the other species) are key to habitat type identification and fragmentary records completion (even more than the exact cover-abundance information of each individual species). When surveying plant species, it might be hard, whatever the level of expertise, to accurately collect the exact abundance of plants in a vegetation plot. However, recording the relative abundance of the most abundant species is much easier and often sufficient. It has to be noted that we did not explicitly consider the spatial scale when selecting data for domain adaptation (the fill-mask task) and training (the text classification task). Since plant species typically co-occur at small spatial scales (a few metres), including plots from larger spatial scales may introduce noise by grouping species that do not actually form a coherent community. For example, a few metres’ difference in elevation or soil moisture can lead to entirely different plant communities, yet a model trained on large-scale data may incorrectly associate species that do not truly co-occur. The larger the spatial scale used, the messier the ecological signal becomes. We did not account for this explicitly because EVA contains a limited number of plots, and we aimed to retain as many as possible, assuming that vegetation scientists conducted relevés with spatial scale in mind. However, future work should investigate how different spatial resolutions impact model performance.

The use of LLMs for understanding vegetation patterns is particularly interesting because these models can learn and interpret the syntax of plant species assemblages. As natural languages are composed of words following grammatical rules, plant assemblages can be thought of as following certain ecological ‘rules’ that dictate how species co-occur and interact^49^. By leveraging the bidirectional architecture of BERT, Pl@ntBERT can effectively learn these intricate patterns by capturing relationships between species in both forward and backward directions, which provides a more comprehensive view of assemblage composition. This allows the model to understand not only direct associations but also higher-order dependencies within complex assemblages^50^. Such a syntactic approach enables Pl@ntBERT to represent ecological interdependencies with a level of detail that is challenging for traditional statistical methods, offering an alternative way of encoding the relationships that define biodiversity. Through this perspective, Pl@ntBERT provides a more nuanced understanding of the ‘grammar’ underlying ecosystem composition and dynamics, which could support better conservation and habitat management strategies, and possibly a better fundamental understanding of nature. However, as it is an LLM, Pl@ntBERT can only learn from existing datasets and cannot anticipate novel species assemblages that may emerge in response to climate change, species invasions or land-use changes. This is particularly relevant for neoecosystems, where new combinations of native and non-native species form as environmental conditions shift. Pl@ntBERT cannot infer future biodiversity patterns beyond what is already recorded in datasets, meaning that ongoing field surveys and expert input remain essential. Ecologists will need to continuously document new assemblages and update training data to keep the model relevant in a rapidly changing world. This underscores that Pl@ntBERT is not a replacement for field expertise but rather a tool to assist researchers in making sense of complex ecological patterns.

When it comes to vegetation classification, having a good understanding of how and why Pl@ntBERT assigns a EUNIS habitat type to a given vegetation plot is essential if we want researchers and practitioners to trust the results^51^. Integrated gradients^52^, a method to calculate how important each input feature (that is, plant species) is to the prediction (that is, habitat types), were used to explain how positively or negatively a species contributes to the classification of a vegetation plot. A more detailed overview of species attributions on a vegetation plot can be found in appendix 28 in the Supplementary Information. It is interesting to see how a change in diagnostic, constant or dominant taxa can change the model behaviour. This study shows that the most abundant species in a vegetation plot (that is, the first species of the sentence) is often the one that contributes the most to the classification, which reflects the experience with probabilistic keys for identifying vegetation types^53^. One of the advantages of this model is that it brings vegetation science closer to a wider circle of people.

Other experiences, whose details can be found in appendix 22 in the Supplementary Information, corroborate these findings. When the information on abundance is removed (that is, by forming sentences with species in random order), the performance of Pl@ntBERT significantly drops. For example, the accuracy of the text classification task decreased by 14% compared with the classical approach. This drop was more substantial than when we kept the information on abundance but removed 30% of the species by random selection, meaning that capturing the relative abundance is more important than recording all plant species. Similarly, when it comes to finding which species is hiding behind a mask in a vegetation plot, Pl@ntBERT went from assigning the correct species in over 17% of the cases when the species were sorted to less than 7% of the cases when the species were not sorted. This means that plant assemblages are defined not only by the species present but also by their order of abundance because abundance influences community structure, ecological interactions and ecosystem functioning. Abundance also influences functional diversity, which is critical for ecosystem processes. Species with higher abundance often have significant roles in ecosystem functioning due to their traits and interactions with other species^54^.

While Pl@ntBERT demonstrates promising results in identifying vegetation patterns and assigning habitat types on the basis of species co-occurrence, one key limitation of the current model is that it does not explicitly account for the vertical structure of plant communities. Some habitats are characterized not only by their species composition but also by their layering structure, which plays a crucial role in defining their ecological identity. Thus, a possible improvement would be to introduce explicit hierarchical encoding of vegetation strata in Pl@ntBERT’s input data. This could be achieved by adopting a standardized syntax, such as: ‘Tree layer: *Fagus sylvatica*, *Quercus robur*; Shrub layer: *Carpinus betulus*, *Fagus sylvatica*, *Corylus avellana*; Herb layer: *Anemone nemorosa*, *Hyacinthoides non-scripta*, *Mercurialis perennis*’. If layering information is integrated into Pl@ntBERT’s training, the model could better capture functional differences between habitats (especially those that are defined as much by their structural complexity as by species composition alone), improve classification accuracy and potentially enhance its ability to predict missing species within specific strata. This hierarchical representation could also facilitate better interpretability, as users could analyse species associations within distinct vertical layers rather than treating all species as equally co-occurring in a single homogeneous space. Future work should explore how to best format and standardize stratification data, as well as whether habitat-specific differences in layering (for example, grasslands versus forests) require different encoding strategies. Incorporating structural information into Pl@ntBERT could significantly refine its ecological modelling capabilities, making it a more powerful tool for vegetation science and conservation applications.

The study areas of the experiments done with Pl@ntBERT were Europe and adjacent areas (for example, Anatolia and the Caucasus). A key challenge for scaling the model beyond the studied regions lies in its transferability to undersampled or floristically distinct areas. While our model was trained on a large and diverse corpus of vegetation plots across Europe, applying it to other biogeographic regions will require retraining or fine-tuning on locally relevant species assemblages. However, data scarcity in such areas could limit the model’s performance. One possible solution lies in leveraging transfer learning, where Pl@ntBERT could be adapted to new regions using smaller, region-specific datasets (for example, the Tropical African Vegetation Archive, which is a continental data aggregator, similarly to EVA) while retaining general semantic knowledge from the broader model. Future work could explore this idea by subsampling existing training data or simulating low-data settings to evaluate performance degradation and retraining requirements. Such studies are essential to assess the robustness and practical deployment of LLMs for global biodiversity monitoring.

Beyond habitat identification and assemblage completion tasks, Pl@ntBERT may also provide insights into ecosystem condition by detecting deviations from the expected species assemblages. For instance, by comparing the real observed species lists to the model’s predicted co-occurrence patterns, it is possible to quantify how ‘natural’ a given community appears. Such deviations could reflect ecological disturbances, including invasion by non-native species. In particular, we foresee applications in early warning systems where the increasing dominance of introduced species might signal ecosystem change. In this context, the likelihoods of the masked species output by Pl@ntBERT in given assemblages could serve as indicators of unexpected patterns, complementing traditional biodiversity indicators. For instance, starting from an observed assemblage, masking each species one by one would give the likelihood of the species belonging to the assemblage (that is, the higher the value, the more expected the species within the assemblage). As a result, counting the number of species below a given threshold (that is, through a statistical hypothesis test) in an assemblage could give an insight on the habitat condition (for example, by detecting the increasing dominance of non-native species). Exploring such diagnostic capabilities represents a promising direction for future research.

While the masked species prediction task was primarily designed to evaluate the model’s understanding of species co-occurrence patterns, we acknowledge that its direct use in field applications may be limited. However, it opens up interesting possibilities for developing alternative field protocols. For example, one could imagine adaptive sampling schemes where species discovery rates are tracked, and sampling is stopped once a threshold is reached (for example, around 15 species, more or less depending on the habitat type and plot size). A model such as Pl@ntBERT could then suggest additional likely, but unobserved, species on the basis of the partial list, particularly focusing on less abundant or harder-to-detect taxa. Such an approach could accelerate vegetation surveys while incorporating the model’s uncertainty. More broadly, we see this task as a conceptual bridge towards expert-led and model-assisted field methods, where machine learning can help (but not replace) vegetation experts.

As a perspective, one promising direction is to use Pl@ntBERT to complete partial species assemblages derived from species distribution models (SDMs). While SDMs have long been used to predict species occurrences on the basis of environmental conditions^55^, recent deep-learning-based approaches, referred to as deep-SDMs, have shown stronger performance for modelling vascular plant species distribution^56–59^. Typically, these models generate a ranked list of species predicted to occur at a given location. Pl@ntBERT could be applied to such lists to infer plausible co-occurring species that might have been missed or are underreported, especially in the context of citizen science observations.

Citizen science platforms^60,61^ now provide far more plant occurrence data than traditional vegetation-plot databases^62^. However, these data often lack completeness, as contributors tend to report only common or iconic species and may miss rarer or harder-to-identify taxa^63,64^. Here, Pl@ntBERT could be particularly useful: by capturing co-occurrence patterns learned from expert-labelled vegetation plots, it can be used to fill in likely missing species and improve the quality of predicted assemblages. Within citizen science platforms, such as Pl@ntNet or iNaturalist, Pl@ntBERT could help with automated species identification by estimating which species are most likely on the basis of those already observed in an area.

More broadly, a future pipeline could combine multiple deep learning techniques to build habitat distribution models. For instance, image classification models (for example, convolutional neural networks^65^) could be used to extract environmental features from satellite imagery and predict likely species occurrences. Pl@ntBERT could then apply a fill-mask strategy to reconstruct plausible assemblages from these partial species lists. Finally, Pl@ntBERT could again be used, this time to assign habitat types using text classification on the predicted assemblages. Such a multimodal and end-to-end approach^66^ could bridge the gap between raw species occurrence data and habitat type inference, contributing to finer-scale and more scalable biodiversity monitoring.

## Methods

A visualization of the methodology used in this paper is shown in Fig. 1, a more complete overview is provided in appendix 26 in the Supplementary Information and a detailed description of each step is shown in Supplementary Figs. 9–11. An explanation of all acronyms and terms can be found in Supplementary Texts 30 and 31.

### Leveraging vegetation plots

The data used for training the Pl@ntBERT model were extracted from EVA^37^. EVA is a database of vegetation plots—that is, records of plant taxon co-occurrence that have been collected by vegetation scientists at particular sites and times. The EVA data were extracted on 22 May 2023. They contained all georeferenced plots from Europe and adjacent areas (that is, 1,731,055 vegetation plots and 36,670,535 observations from 34,643 different taxa).

These vegetation plots were first split into two sets, depending on the presence or absence of a habitat type label:

1. A dataset containing unlabelled data—that is, vegetation plots with a missing indication of EUNIS habitat type. This dataset (henceforth ‘fill-mask dataset’) containing 572,231 vegetation plots could be used only for training the masked language model.
2. A dataset containing labelled data—that is, vegetation plots with an indication of EUNIS habitat type. This dataset (henceforth ‘text classification dataset’) containing 850,933 vegetation plots could be used for training both the masked language model and the text classification model.

To ensure a clean dataset representing vegetation patterns well, some additional pre-processing steps were conducted. We removed the few species with a given cover percentage of 0, assuming these were errors or scientists reporting absent species (which resulted in 31,813,043 observations remaining). We merged duplicated species in the same vegetation plots (that is, species that appeared twice or more in one vegetation plot because they were in different layers) and summed their percentage covers (which resulted in 31,036,661 observations remaining). The taxon names were then standardized using the API of the Global Biodiversity Information Facility (GBIF). It relies on the GBIF Backbone Taxonomy as its nomenclatural source for species taxon names and integrates and harmonizes taxonomic data from multiple authoritative sources (for example, Catalogue of Life, International Plant Names Index and World Flora Online). As EVA is an aggregator of national and regional vegetation-plot databases, this step ensured that the same species collected in two very distant areas still shared the same name^67^. If no direct match was found for the species name (for example, the GBIF Backbone Taxonomy was not able to provide a scientific name for the EVA species *Carex cuprina*), then it was dropped. As we focused on the species taxonomic rank, taxa identified only to the genus level were dropped, and taxa identified at the subspecies level were lumped together at the species level (for example, *Hedera* was dropped but both *Hedera helix* subsp. *helix* and *Hedera helix* subsp. *poetarum* were merged into *Hedera helix*). This resulted in 29,859,407 observations remaining. We removed hybrid species and very rare species (that is, species that appeared less than ten times in the whole dataset), which resulted in 29,836,079 observations remaining. Vegetation plots that lost more than 25% of their taxa or their most abundant taxon after the species name matching were removed from the dataset, to ensure that the remaining plots still provided reliable representations of vegetation patterns (which resulted in the final number of 29,149,022 observations remaining). Finally, vegetation plots belonging to very rare habitat types (that is, habitat types that appeared less than ten times in the whole dataset) were considered unlabelled data and added to the fill-mask dataset.

**Fig. 5:**
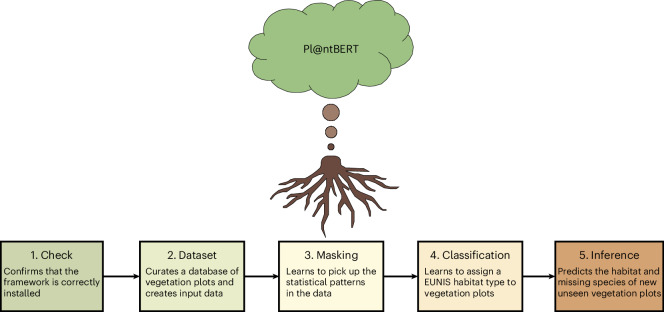
Overview of the framework. The sequence of tasks performed during each of the five main stages (installation check, dataset curation, masking training, classification training and outcome prediction).

The set of labelled vegetation plots was then strategically split. To avoid overfitting, ideally part of the available labelled data must be held out as a test set. However, the quantity of available full lists of plant species with estimates of cover abundance of each species and habitat type assignment is not very high (that is, less than 1,000,000 vegetation plots for all of Europe, a relatively low number compared with the vast amount of biodiversity data available). Partitioning the available data into a training set and a test set would reduce the number of training samples to a level too low for effective model training. It is therefore possible to use *k*-fold cross-validation to split the dataset into *k* subsets instead. Then, for each of the splits, the model can be trained using *k* − 1 of the subsets for training and the latter one for validation. However, cross-validation scores for the classification of vegetation plots can be biased if the data are randomly split, because they are commonly spatially autocorrelated (spatially closer data points have similar values). One strategy to reduce the bias is splitting data along spatial blocks^68^. This procedure avoids fitting structural patterns and allows the separation of near-duplicates. Such vegetation plots differ from each other in a very small portion of species (for example, if they are close in space, two vegetation plots may exhibit identical plant composition but feature species with slightly contrasting abundances). The dataset was thus first split into spatial blocks of 6 arcmin (0.1° on the World Geodetic System 1984 spheroid). The blocks were then split into folds. Since the geographic distribution of vegetation plots across Europe is unequal, each block can have a different number of data points. The folds were thus balanced to have approximately equal numbers of plots instead of assigning the same number of blocks to each fold (which could have led to folds with very different numbers of data points). This process was facilitated by the use of the research software Verde.

With over 1,400,000 vegetation plots, 29,000,000 observations and 14,000 species, the dataset used in this paper is one of the most extensive datasets of vegetation plots ever analysed^69^. The entire description of the dataset can be found in Supplementary Table 2, and a visualization of the data can be found in appendix 32 in the Supplementary Information. An overview of the long-tail distribution of species (that is, there is a strong class imbalance, meaning that a few species are present in many of the vegetation plots) can be found in Supplementary Fig. 14, and more taxonomic information on the species (for example, class, order and family), mostly vascular plants with some bryophytes and lichens, can be found in appendix 16 in the Supplementary Information.

The EUNIS habitat types^18^ are referred by their codes instead of their names, as they better reflect the classification hierarchy. The coding system is structured so that each broad habitat group is represented by one letter (except the broad habitat group littoral biogenic habitats, which is designated by the code MA2). A new alphanumeric character is then added for each subsequent level. For instance, the habitat type Mediterranean, Macaronesian and Black Sea shifting coastal dune is identified by the code N14, indicating its belonging to the habitat group N1 (that is, coastal dunes and sandy shores), and more generally to the broad habitat group N (that is, coastal habitats). The entire list of the 227 habitat types used in this work can be found in appendix 24 in the Supplementary Information, but to exemplify the habitat types included, we list the eight broad habitat groups used in this paper below:

- Littoral biogenic habitats (code: MA2)—11 habitat types belonging to littoral habitats formed by animals such as worms and mussels or plants (salt marshes)
- Coastal habitats (code: N)—25 habitat types belonging to habitats above the spring high tide limit (or above the mean water level in non-tidal waters) occupying coastal features and characterized by their proximity to the sea, including coastal dunes and wooded coastal dunes, beaches and cliffs
- Wetlands (code: Q)—17 habitat types belonging to wetlands, with the water table at or above ground level for at least half of the year, dominated by herbaceous or ericoid vegetation
- Grasslands and lands dominated by forbs, mosses or lichens (code: R)—52 habitat types belonging to non-coastal land that is dry or only seasonally wet (with the water table at or above ground level for less than half of the year) with greater than 30% vegetation cover
- Heathlands, scrub and tundra (code: S)—42 habitat types belonging to non-coastal land that is dry or only seasonally inundated (with the water table at or above ground level for less than half of the year), usually with greater than 30% vegetation cover and with the development of soil
- Forests and other wooded land (code: T)—45 habitat types belonging to land where the dominant vegetation is, or was until very recently, trees with a canopy cover of at least 10%
- Inland habitats with no or little soil and mostly with sparse vegetation (code: U)—23 habitat types belonging to non-coastal habitats on substrates with no or little development of soil, mostly with less than 30% vegetation cover, that are dry or only seasonally wet (with the water table at or above ground level for less than half of the year)
- Vegetated man-made habitats (code: V)—12 habitat types belonging to anthropogenic habitats that are dominated by vegetation and usually subject to regular management but also arising from recent abandonment of previously cultivated ground

The final dataset created solely for the fill-mask task (that is, the fill-mask dataset) contained a total of 572,231 vegetation plots covering 14,069 different species. This dataset of 10,853,856 species observations (on average 19 species per plot) was used only for fine-tuning the masked language model, as each sample was unlabelled (the vegetation plots in this set were not classified to a habitat type). Each sample was used for the fill-mask task during each split in the training set, along with around 90% of the text classification dataset.

The text classification dataset, which was created for both the fill-mask task and the text classification task, contained a total of 850,933 vegetation plots covering 13,727 different species. This dataset of 18,295,166 species observations (on average around 22 species per plot) was used for fine-tuning the masked language model and for training the classifier head (that is, the module added on top of the masked language model to transform its outputs into predictions for assigning habitat types to vegetation plots), as each sample was labelled (the vegetation plots in this set were classified to a habitat type). Each sample was used nine times in the training set and once in the test set.

### Pl@ntBERT fill-mask model training

Every plant species has specific environmental preferences that shape its presence. The task of masking some of the species in a vegetation plot and predicting which species should replace those masks can therefore help get a good contextual understanding of an entire ecosystem. This process is known as fill-mask. A detailed description of the hardware used to train the models can be found in Supplementary Text 3.

Pl@ntBERT is based on the vanilla transformer model BERT^36^. Hence, to predict a masked species in a vegetation plot, the model can consider (that is, focus on and process information using the attention mechanism in the transformer architecture) all species bidirectionally. This means that the model, when looking at a specific species, has full access to the species on the left (that is, more abundant species) and right (that is, less abundant species). The two original BERT models (that is, base and large) were leveraged for this study. BERT-base has 12 transformer layers (that is, transformer blocks) and 110,000,000 parameters (that is, learnable variables), and BERT-large has 24 transformer layers and 340,000,000 parameters. A detailed description of the architecture of the two sizes can be found in Supplementary Table 1. Moreover, the uncased version of BERT was leveraged to train Pl@ntBERT. This version does not distinguish between ‘*hedera*’ and ‘*Hedera*’. Hence, as all outputs from Pl@ntBERT would be in lowercase, all inputs (abundance-ordered plant species sequences) were also lowercased to ensure consistency. For these two reasons, each sentence fed into the model was formed by listing all the species in descending abundance order, in lowercase and separated by commas. When species had the same cover (which is frequent as most EVA data come from ordinal scales with a few steps only), they were randomly ordered.

For many natural language processing applications involving transformer models, it is possible to simply take a pretrained model and fine-tune it directly on some data for the task at hand. Provided that the dataset used for pretraining is not too different from the dataset used for fine-tuning, transfer learning will usually produce good results. The predictions depend on the dataset the model was trained on, since it learns to pick up the statistical patterns present in the data. However, our dataset contains binomial names (that is, the scientific names given to species and used in biological classification, which consist of a genus name followed by a species epithet). Because it has been pretrained on the English Wikipedia and BookCorpus datasets, the predictions of the vanilla transformer model BERT for the masked tokens will reflect these domains. BERT will typically treat the species names in the dataset as rare tokens, and the resulting performance will be less than satisfactory. By fine-tuning the language model on in-domain data, we can boost the performance of the downstream task. This process of fine-tuning a pretrained language model on in-domain data is called domain adaptation. Vegetation-plot records from EVA that were not assigned to a habitat type were used for this task. The sentences were created by ordering each species within a plot in descending order of abundance, separating them by commas. Two different ways were used to tokenize (that is, prepare the inputs for the models) the names of the species:

1. The ‘term’ way: a species name is divided into two tokens, one for the genus name and one for the species epithet.
2. The ‘species’ way: a whole binomial name is equivalent to a token.

More information about the versions of Pl@ntBERT can be found in Supplementary Table 7. For each approach, two model sizes were leveraged: base and large.

Unlike other natural language processing tasks, such as token classification or question answering, where a labelled dataset to train on is given, there are not any explicit labels in masked language modelling. A good language model is one that assigns high probabilities to sentences that are grammatically correct and low probabilities to nonsense sentences. Assuming our test dataset consists of sentences that are coherent plant assemblages, one way to measure the quality of our language model is to calculate the probabilities it assigns to the masked species in all the sequences of the test set. High probabilities indicate that the model is not ‘surprised’ or ‘perplexed’ by the unseen examples (that is, describing the model’s uncertainty or difficulty in predicting masked elements, hence reflecting how well it has learned the underlying structure of the data) and suggests it has learned the basic patterns of grammar in the language (in the case of Pl@ntBERT, the language being ‘floristic composition’). As a result, the perplexity, which is defined as the exponential of the cross-entropy loss, is one of the most common metrics to measure the performance of language models (the smaller its value, the better its performance). It was used in our experiments to evaluate the model in addition to the species masking accuracy.

Except for commas, the classify tokens [CLS] (which represent entire input sequences) and the separate tokens [SEP] (which mark the separation between different input sequences), 15% of the tokens were ‘masked’ during the experiments. These tokens consisted of full species names in the case of Pl@ntBERT-species and of genus names or species epithets in the case of Pl@ntBERT-term. We followed the same procedure used in the original BERT paper^36^: each selected token was replaced by (1) the [MASK] token 80% of the time, (2) a random species 10% of the time or (3) the same species 10% of the time. Each model was trained for five epochs (that is, five complete passes of the training dataset through the model). This process was facilitated by the use of the deep learning package Pytorch^70^ and the open-source library HuggingFace^71^.

To compare how Pl@ntBERT models species assemblages compared to traditional approaches, we also implemented three alternative baseline methods solely based on species co-occurrence information. The first one is a version of Pl@ntBERT for which species are given as input in random order rather than abundance-ordered. This makes it possible to remove the information linked to the order of species so that most of the syntax rules cannot be learned anymore apart from co-occurrence patterns. The second baseline method is a naive Bayes predictor based on the species co-occurrence matrix. Ten different co-occurrence matrices were built, each time leveraging all the dataset minus one fold (to always keep the ground truth hidden). As a result, each matrix indicates how many times species of each pair co-occur in the same vegetation plots in the nine training folds. From the co-occurrence matrix, we can derive the probability of each species conditionally to an observed species assemblage. More details about how this naive Bayes predictor is constructed can be found in Supplementary Equation (25). The last baseline method is a neural network optimizing the log-loss function using stochastic gradient descent. It was trained on incomplete species assemblages (that is, for every vegetation plot of the training set, a species was randomly masked, and the goal of the model was to retrieve it). More details about how the multilayer perceptron is implemented can be found in appendix 21 in the Supplementary Information.

### Identifying habitat types

The classification of vegetation provides a useful way of summarizing our knowledge of vegetation patterns. The task of assigning a habitat type to sentences describing floristic compositions therefore serves to describe many facets of ecological processes. This process is called text classification.

Pl@ntBERT is based on the fine-tuned version of BERT, meaning it has already adapted its weights to predict species that are more strongly associated with the plants from the sentence. It provides a better foundation for learning task-specific models, such as a text classification model. To create a state-of-the-art model for vegetation classification, we added one additional output layer (that is, a fully connected layer that matched the number of habitat types) on top of the pooled output.

Vegetation-plot records from EVA that were assigned to a habitat type were used for this task. The habitat labels were generated using the expert system EUNIS-ESy v.2021-06-01 (ref. ^19^) directly by the coordinators of the EVA database using the JUICE program. This means that using EUNIS-ESy to identify the habitat types of the raw data from EVA (without the pre-processing steps such as harmonizing the taxon names) should lead to an accuracy of 100%. Each model was trained for five epochs.

To evaluate the classification performance, we computed accuracy, precision, recall and F1-score on the test set. Given the class imbalance in habitat labels (for example, the habitat type R22 (that is, low and medium altitude hay meadow) is present 69,533 times in the text classification dataset, and the habitat type U35 (that is, boreal and arctic base-rich inland cliff) is present 12 times in the text classification dataset), the F1-score was particularly useful in assessing how well the model performed across different habitat types. We also compared Pl@ntBERT’s performance against a standard BERT model trained from scratch on the same dataset to assess the benefits of domain adaptation. Finally, we compared the results with EUNIS-ESy and hdm-framework, respectively a classification expert system and a deep-learning framework.

### Inclusion and ethics

This study is based on vegetation-plot data sourced from EVA, a collaborative effort that aggregates vegetation data from across Europe and neighbouring regions. The data used in this study come from 110 EVA member databases, with permissions granted by individual data custodians (listed in appendix 35 in the Supplementary Information).

- Local collaboration and roles: The data used in this study were collected and curated by a wide network of local researchers. Each dataset included was used with explicit permission from its respective custodian, who retains data ownership. Co-authorship was offered to at least one representative of each database who was interested in the project and willing to intellectually contribute to the study, hence including local researchers in this process.
- Local relevance and co-design: The research aims to understand large-scale patterns in plant biodiversity and vegetation structure, which is directly relevant for regional and continental conservation planning, habitat classification and biodiversity monitoring. Including local partners in the design of the specific research questions and using their datasets representing decades of local ecological research was foundational to the project.
- Ethical review and approvals: Since the study involves secondary analysis of existing vegetation data, no local or institutional ethics board approval was required. The EVA data policy governs the ethical use of contributed data, and no sensitive or identifiable information is included.
- Compliance with local regulations and standards: All original data collection followed the environmental, legal and ethical standards of the respective countries where plots were sampled. The study complies with the EVA framework, which ensures that data use respects both the legal and ecological context of data origin.
- Risk and harm considerations: The research does not involve human or animal subjects and poses no risk of stigmatization, incrimination or discrimination. There are no safety risks to researchers or participants, and no biological materials, cultural artefacts or associated traditional knowledge were transferred.
- Benefit sharing and capacity building: While the study does not involve new biological sample collection, all results will be shared publicly through this scientific publication and with the EVA data custodians. The project supports the visibility of local contributions by highlighting the role of regional databases and cites local and regional literature where relevant.
- Citations and recognition: The study references and builds on local ecological knowledge that is contained in the EVA datasets, and acknowledges the scientific and curatorial work of local data contributors.

Artificial intelligence tools such as ChatGPT (OpenAI) and Copilot (GitHub) were used to assist in writing the manuscript and coding the framework (Fig. 5), respectively. All outputs were critically reviewed and edited by the authors. See Supplementary Text 37 for a more in-depth explanation.

### Reporting summary

Further information on research design is available in the Nature Portfolio Reporting Summary linked to this article.

## Supplementary information

Supplementary Information Supplementary Figs. 1–19, Tables 1–10 and texts.

Reporting Summary

## Data Availability

The data that support the findings of this study are available from EVA, but restrictions apply to the availability of these data, which were used under licence for the current study and so are not publicly available. The data are, however, available from the authors or EVA custodians upon reasonable request and with the permission of EVA. The DOI of the EVA data selection for this project is 10.58060/QR4B-G979.

## Code availability

The generic, free and open-source framework that supports the findings of this study is available via GitHub at https://github.com/cesar-leblanc/plantbert. See Fig. 5 for an overview of the list of tasks that Pl@ntBERT can achieve.
