# Supplementary material for: Learning the syntax of plant assemblages
Source: Nat Plants. 2025 Oct 13;11(10):2026–40. doi: 10.1038/s41477-025-02105-7 (PMC12537494; doi:10.1038/s41477-025-02105-7)
Supplement: Supplementary file 1 — Supplementary Figs. 1–19, Tables 1–10 and texts. [file 41477_2025_2105_MOESM1_ESM.pdf]

nature plants

Supplementary information https://doi.org/10.1038/s41477-025-02105-7

Learning the syntax of plant assemblages

In the format provided by the authors and unedited

Supporting information to the paper
Leblanc, C., Bonnet, P., Servajean, M., Thuiller, W., Chytrý, M., ... & Joly, A. (2025). Learning the syntax of plant assemblages. In *Nature Plants*.

Contents

S1 - Models 2
S2 - Data 2
S3 - Hardware 2
S4 - Fine-tuning 3
S5 - Results 3
S6 - Metrics 4
S7 - Details 5
S8 - Attention 5
S9 - Split 6
S10 - Masking 7
S11 - Classification 8
S12 - Habitats 9
S13 - Confusion 9
S14 - Species 10
S15 - Syntaxon 10
S16 - Hierarchy 10
S17 - Ablation 11
S18 - Tokenization 11
S19 - Demo 12
S20 - Framework 13
S21 - Parameters 14
S22 - Performance 15
S23 - Examples 15
S24 - Dataset 16
S25 - Co-occurrence 22
S26 - Workflow 23
S27 - Categories 24
S28 - Explainability 24
S29 - Patterns 25
S30 - Acronyms 25
S31 - Terms 25
S32 - Visualization 26
S33 - Comparison 27
S34 - Mapping 28
S35 - Databases 29
S36 - Rank 31
S37 - Assistance 31

S1 - Models

Table 1. Models architecture

Model	# Transformer layers	Hidden size	# Attention heads	# Parameters
bert-base	12	768	12	110M
bert-large	24	1,024	16	340M

S2 - Data

Table 2. Datasets description

Dataset	# Vegetation plots	# Observations	# Species	Unique species	# Habitat types
Fill-mask	572,231	10,853,856	14,069	462	~
Text classification (fold 0)	85,087	1,872,339	10,046	3	219
Text classification (fold 1)	85,076	1,816,113	10,463	4	222
Text classification (fold 2)	85,115	1,794,832	10,201	0	221
Text classification (fold 3)	85,067	1,851,169	10,464	2	217
Text classification (fold 4)	85,065	1,859,368	10,223	4	216
Text classification (fold 5)	85,081	1,816,258	10,160	1	219
Text classification (fold 6)	85,148	1,830,761	10,155	0	217
Text classification (fold 7)	85,082	1,815,530	10,209	1	217
Text classification (fold 8)	85,053	1,825,228	10,303	6	223
Text classification (fold 9)	85,159	1,813,568	10,231	0	220
TOTAL	1,423,164	29,149,022	14,189	~	227

S3 - Hardware

The models were first tuned on any suitable hardware. The tuned models were then evaluated on a HPE DL385 dual-AMD EPYC 7513 @ 2.6GHz (32 cores) node:

- Nvidia A100 - 80GB PCIe GPUs cards
    - 6,912 CUDA cores per card
    - 432 tensor cores per card
    - 80GB of RAM capacity per card
    - Tensor performance peak: 312 TFlops per card
    - FP16 Compute: 78 Tflops per card
    - FP32 Compute: 19.5 Tflops per card
    - FP64 Compute: 9.7 Tflops per card
    - 1,935GB/s GPU memory bandwidth with error correction (ECC)
- 2x gigabit network ports (one connected)
- infiniband EDR card (connected to FDR switch)
- hyperthreading active

S4 - Fine-tuning

Table 3. Summary of the accuracy (%) reached by the models for each learning rate and batch size on the text classification task (results computed on the first cross-validation fold). The bold entries are the best-performing combination for each model.

(a) Pl@ntBERT-base

	Batch size				
Learning rate	2	4	8	16	32
1e-5	90.88	90.51	90.21	89.93	88.95
2e-5	90.87	90.92	90.71	90.59	90.15
3e-5	90.77	90.92	**90.98**	90.95	90.58
4e-5	90.57	90.73	90.91	90.98	90.71
5e-5	0.00	90.73	90.85	90.71	90.80

(b) Pl@ntBERT-large

	Batch size				
Learning rate	2	4	8	16	32
1e-5	90.99	90.92	90.86	90.58	90.67
2e-5	3.87	90.89	90.85	90.97	90.88
3e-5	3.87	9.14	3.87	90.62	90.71
4e-5	3.87	0.43	3.87	3.87	**91.08**
5e-5	3.87	3.87	0.68	3.87	3.87

(c) Pl@ntBERT-base-term

	Batch size				
Learning rate	2	4	8	16	32
1e-5	91.63	91.40	91.06	90.51	89.97
2e-5	91.75	91.73	91.77	91.52	90.99
3e-5	91.68	91.61	91.75	91.72	91.45
4e-5	91.24	91.73	91.71	91.73	91.67
5e-5	91.09	91.51	91.67	**91.82**	91.75

(d) Pl@ntBERT-large-term

	Batch size				
Learning rate	2	4	8	16	32
1e-5	91.73	91.75	91.73	91.61	91.62
2e-5	3.87	3.87	**91.81**	91.80	91.81
3e-5	3.87	3.87	91.70	91.68	91.76
4e-5	3.87	3.87	91.10	0.01	91.72
5e-5	3.87	3.87	3.87	3.87	3.87

(e) Pl@ntBERT-base-species

	Batch size				
Learning rate	2	4	8	16	32
1e-5	92.01	91.88	91.75	91.11	90.51
2e-5	**92.06**	92.01	91.96	91.83	91.50
3e-5	91.84	91.90	92.02	91.89	91.80
4e-5	91.65	91.86	91.90	91.88	91.83
5e-5	21.67	91.80	91.98	91.91	91.99

(f) Pl@ntBERT-large-species

	Batch size				
Learning rate	2	4	8	16	32
1e-5	91.91	91.93	91.93	91.70	91.57
2e-5	3.87	91.93	91.63	91.94	**91.98**
3e-5	3.87	91.85	91.80	91.97	91.88
4e-5	3.87	3.87	3.87	3.87	3.87
5e-5	3.87	3.87	3.87	3.87	3.87

S5 - Results

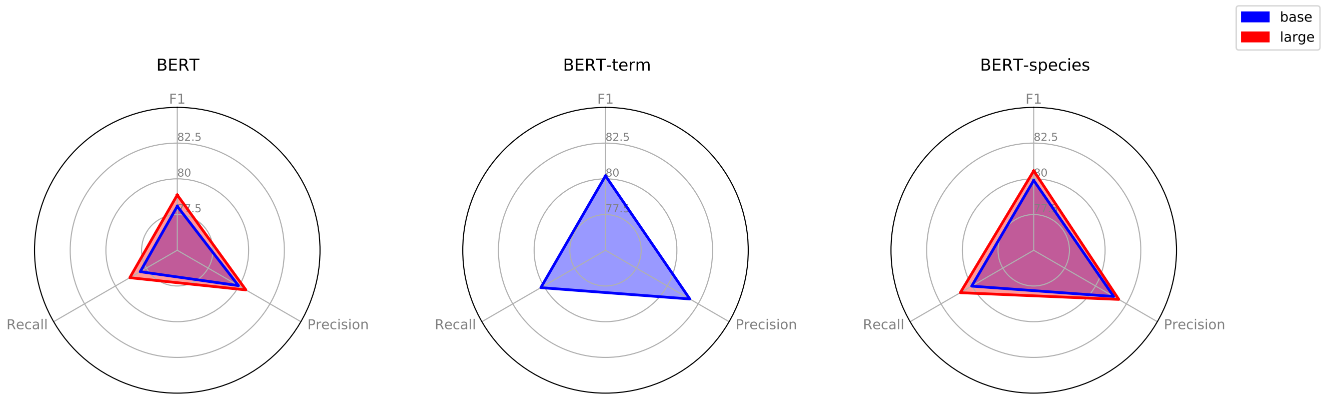

Figure 1. Results reached by all versions of Pl@ntBERT on different metrics (scores macro-averaged over the ten cross-validation folds). Pl@ntBERT-large-term has a F1 of 47.99, a Precision of 48.75 and a Recall of 48.33.

S6 - Metrics

► Micro

Calculate metrics globally by counting the total true positives, true negatives, false positives and false negatives.

► Macro

Calculate metrics for each label, and find their unweighted mean. This does not take label imbalance into account.

► Top-*k*

Number of highest probability or logit score predictions considered to find the correct label.

► Perplexity

Perplexity is the measure of how likely the model is to generate the input text sequence. It is computed via the equation:

$$
\begin{aligned}
PPL(X) &= e^{CE(X)} \\
&= e^{-\frac{1}{t}\log P(X)} \\
&= e^{-\frac{1}{t}\log \prod_{i=0}^{t} p(x_i|x_{<i})} \\
&= e^{-\frac{1}{t}\sum_{i=0}^{t} \log p(x_i|x_{<i})}
\end{aligned} \tag{1}
$$

where $PPL(X)$ is the perplexity of the sample (i.e., how well the language model predicts the sequence), X is the sample (i.e., a sequence of tokens), $CE(X)$ is the cross-entropy of the sample (i.e., how well the model's predicted probabilities match the actual distribution of the next word in the sequence), $P(X)$ is the likelihood of the sample (i.e., the product of each token's probability), t is the length of the sample (i.e., the number of tokens in the sequence) and x is an individual token (i.e., a unit of the sequence).

► Accuracy

Accuracy is the proportion of correct predictions among the total number of samples processed. It is computed via the equation:

$$
\text{Accuracy} = \frac{\text{TP} + \text{TN}}{\text{TP} + \text{TN} + \text{FP} + \text{FN}} \tag{2}
$$

where TP is the number of true positives (i.e., the examples correctly labeled as positive), TN is the number of true negatives (i.e., the examples correctly labeled as negative), FP is the number of false positives (i.e., the examples incorrectly labeled as positive) and FN is the number of false negative (i.e., the examples incorrectly labeled as negative).

► Precision

Precision is the fraction of correctly labeled positive examples out of all of the examples that were labeled as positive. It is computed via the equation:

$$
\text{Precision} = \frac{\text{TP}}{\text{TP} + \text{FP}} \tag{3}
$$

where TP is the number of true positives (i.e., the examples correctly labeled as positive) and FP is the number of false positive (i.e., the examples incorrectly labeled as positive).

► Recall

Recall is the fraction of the positive examples that were correctly labeled as positive. It is computed via the equation:

$$
\text{Recall} = \frac{\text{TP}}{\text{TP} + \text{FN}} \tag{4}
$$

where TP is the number of true positives (i.e., the examples correctly labeled as positive) and FN is the number of false negatives (i.e., the examples incorrectly labeled as negative).

► F1

F1 is the harmonic mean of the precision and recall. It is computed via the equation:

$$
\text{F1} = 2 \times \frac{\text{precision} \times \text{recall}}{\text{precision} + \text{recall}} \tag{5}
$$

S7 - Details

Table 4. Summary of fine-tuning details for the various BERT models used in the paper. All models have a base version and a large version. As the number of added tokens (slightly) varies among each fold, we report here the values of the first split.

Model	Original dataset	# Original tokens	Fine-tuning	Added dataset	# Added tokens	Category of added tokens
BERT	Wiki + Books	30,522	~	~	~	~
BERT-term	Wiki + Books	30,522	fill-mask	EVA	9,367	genus + epithets
BERT-species	Wiki + Books	30,522	fill-mask	EVA	14,186	binomial names

S8 - Attention

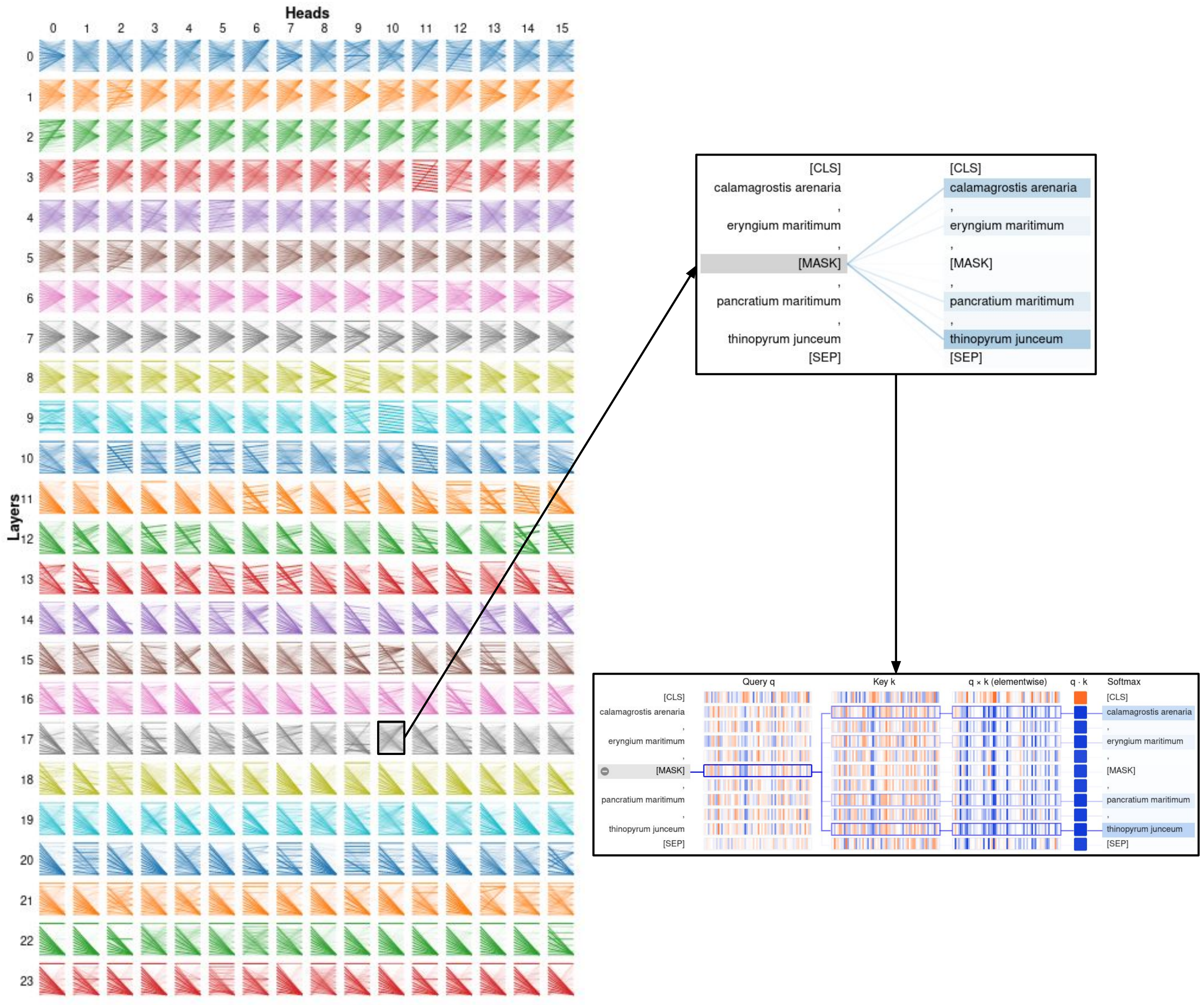

Figure 2. Model (left), head (top-right) and neuron (bottom-right) views for a given input sentence (masked token: *medicago marina*). As the sample belongs to fold 0, the attention was computed using the Pl@ntBERT-large-species model trained on folds 1-9. For the model view, a birds-eye view of attention across all layers and heads is shown. For the head view, connecting lines weighted based on attention between the tokens are shown. For the neuron view, positive and negative values (colored blue and orange, respectively, with color saturation based on magnitude) are shown.

S9 - Split

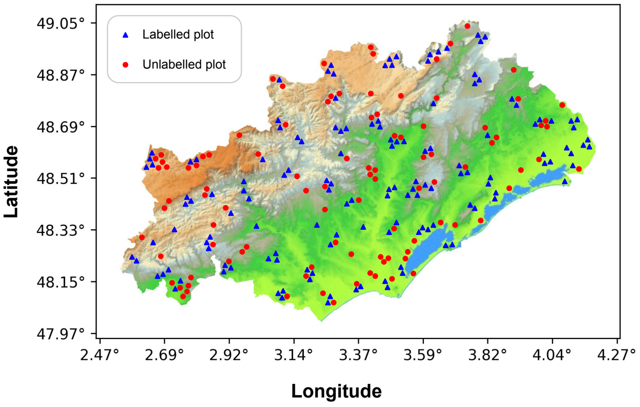

(a) Virtual representation of the curated vegetation plots from EVA (both with and without habitat types assigned). Only the labeled plots are used in both tasks (and both in training and validation).

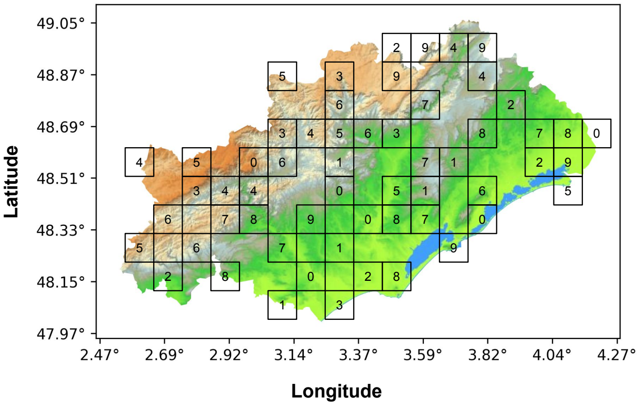

(b) Labeled vegetation plots grouped into rectangular blocks of 6 arc-minutes and split into folds (in a way that makes each fold have approximately the same number of data points).

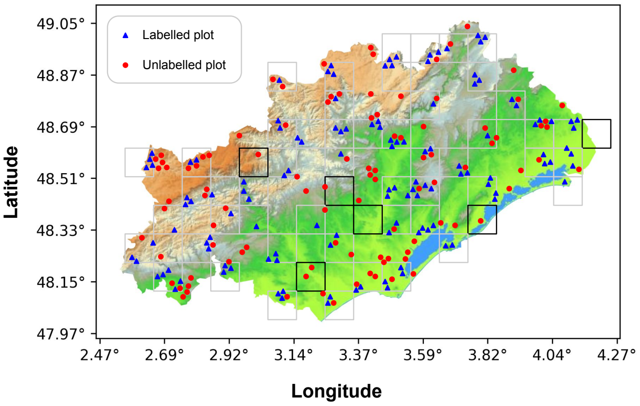

(c) First training set for the fill-mask task (folds 1-9). The training set contains all the unlabeled vegetation plots and the labeled vegetation plots from folds 1-9 (i.e., those in gray blocks).

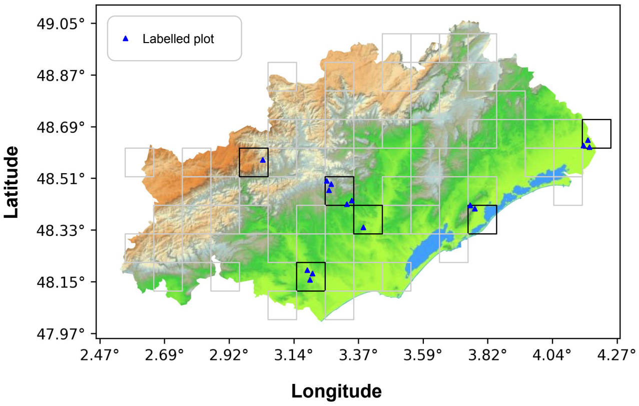

(d) First testing set for the fill-mask task (fold 0). The testing set contains no unlabeled vegetation plot and the labeled vegetation plots from fold 0 (i.e., those in black blocks).

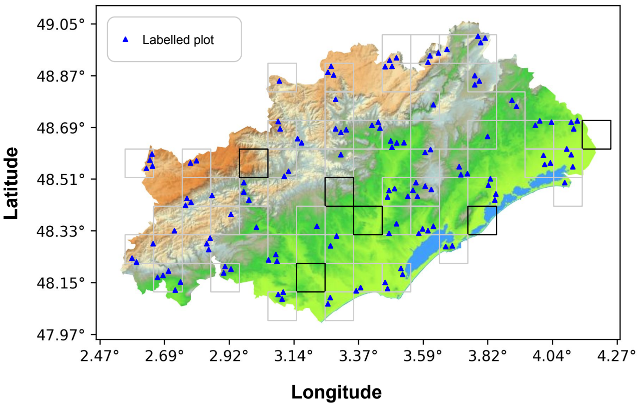

(e) First training set for the text classification task (folds 1-9). The training set contains no unlabeled vegetation plot and the labeled vegetation plots from folds 1-9 (i.e., those in gray blocks).

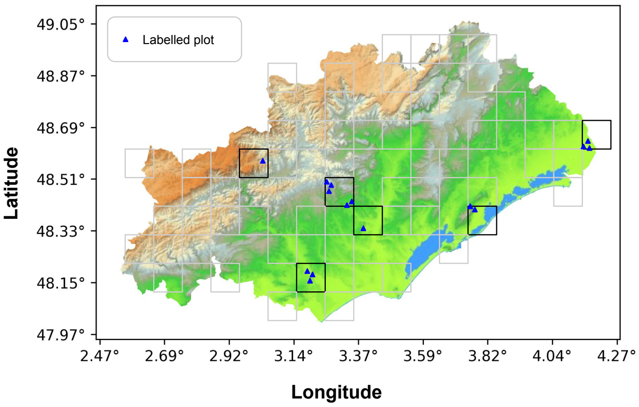

(f) First testing set for the text classification task (fold 0). The testing set contains no unlabeled vegetation plots and the labeled vegetation plots from fold 0 (i.e., those in black blocks).

Figure 3. Spatial split performed during the data curation with a virtual example.

S10 - Masking

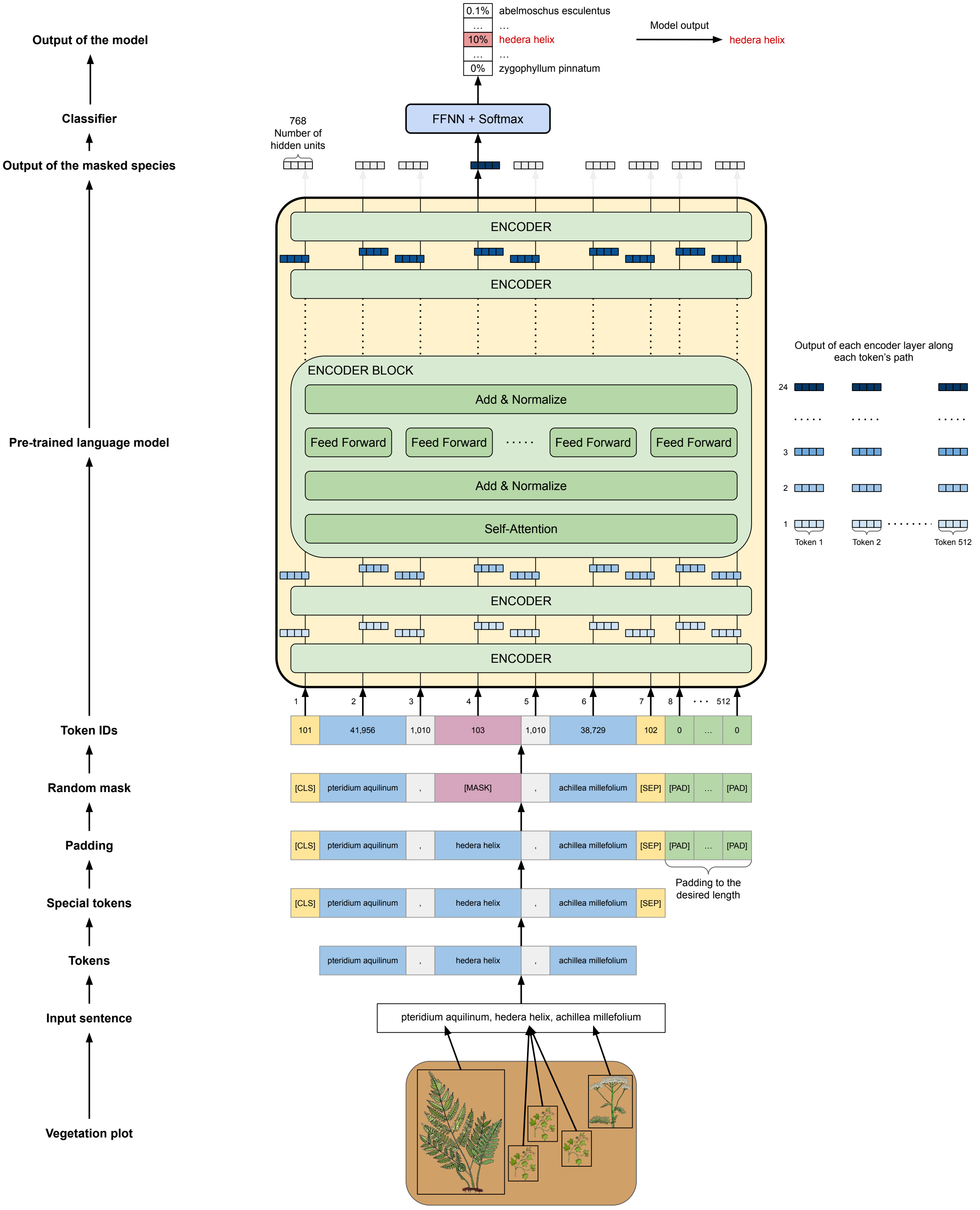

Figure 4. Fill-mask task performed during the fine-tuning of the Pl@ntBERT-large-species models.

S11 - Classification

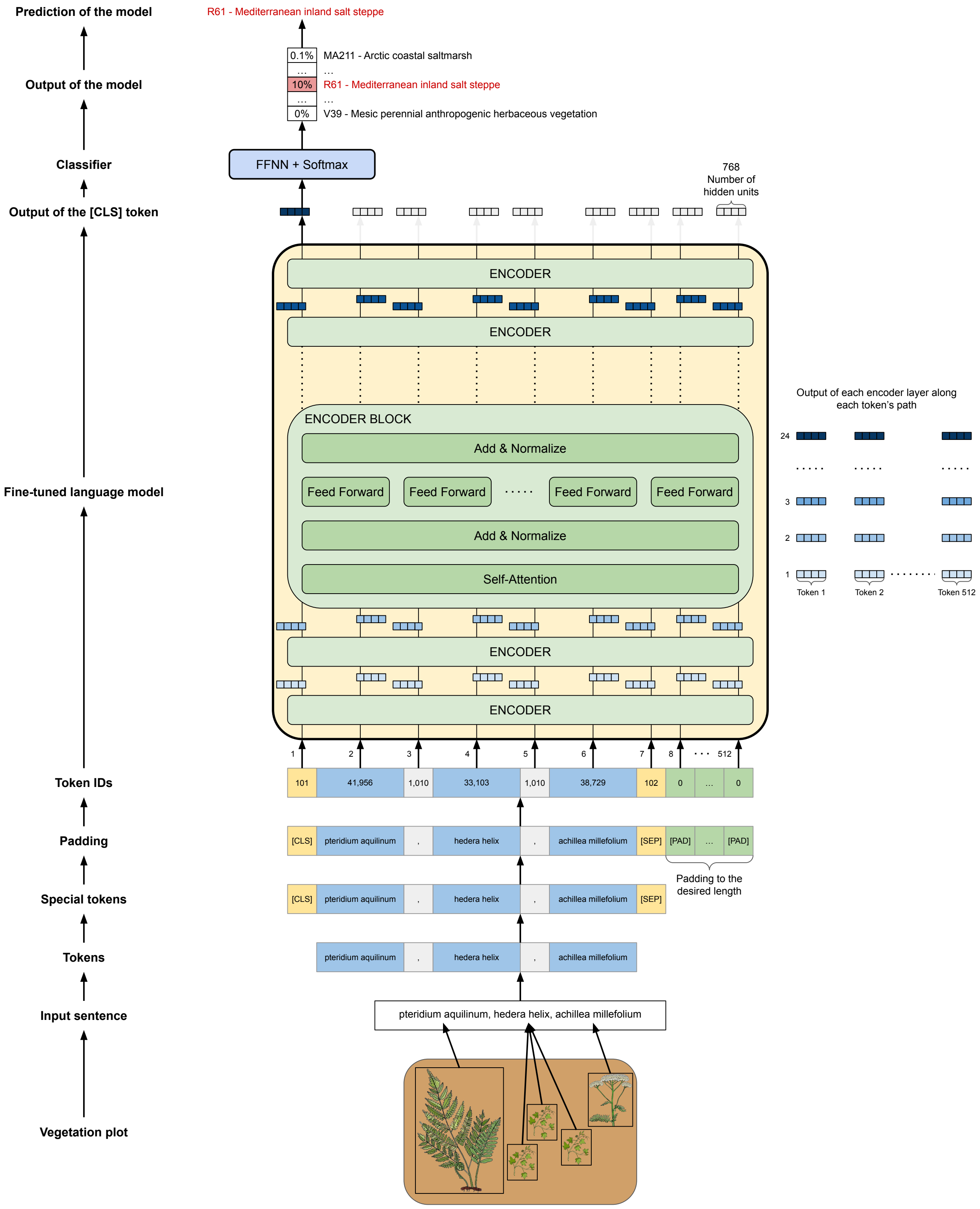

Figure 5. Text classification task performed during the fine-tuning of the Pl@ntBERT-large-species models.

S12 - Habitats

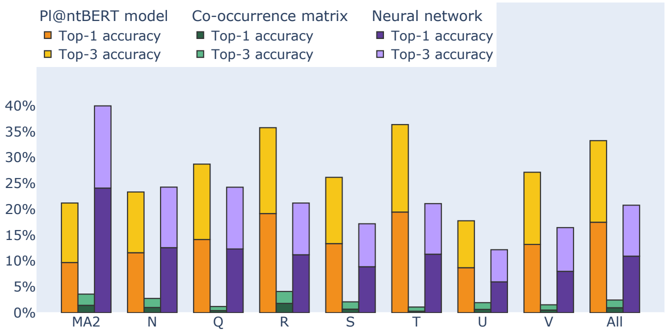

Figure 6. Comparison of Pl@ntBERT, the co-occurrence matrix, and the neural network on the fill-mask task (MA2: Marine, N: Coastal, Q: Wetlands, R: Grasslands, S: Heathlands, T: Forests, U: Inland, V: Man-made). For the purpose of this study, only vegetation plots containing ten species or more were retained (705,479 samples out of the 850,933 samples from the text classification dataset) and exactly one species was (randomly) masked in each sample.

S13 - Confusion

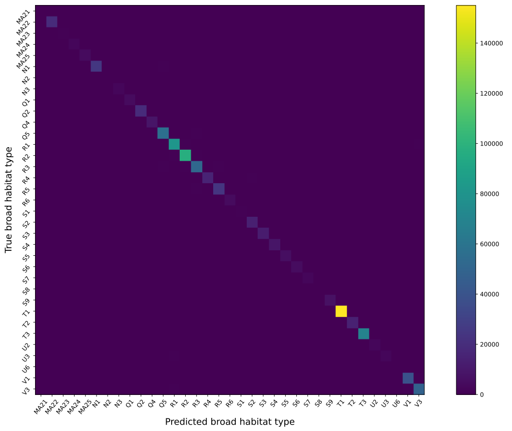

(a) No normalization

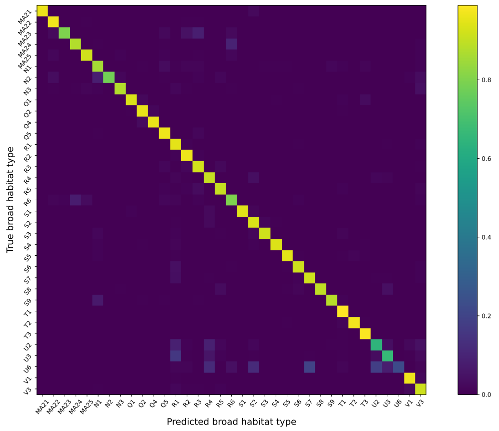

(b) Normalization over the true conditions

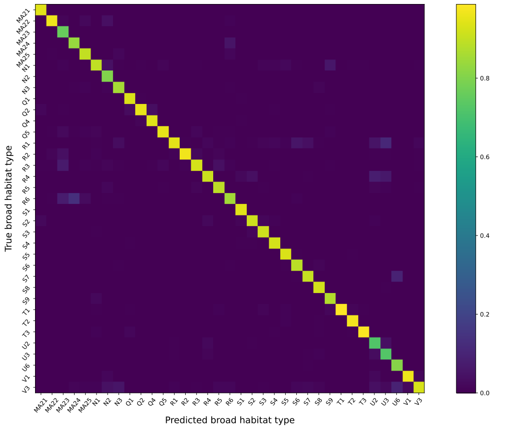

(c) Normalization over the predicted conditions

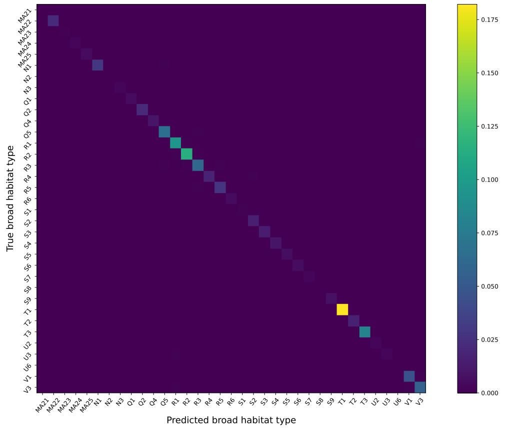

(d) Normalization over all the population

Figure 7. Confusion matrices obtained using the Pl@ntBERT-large-species model

S14 - Species

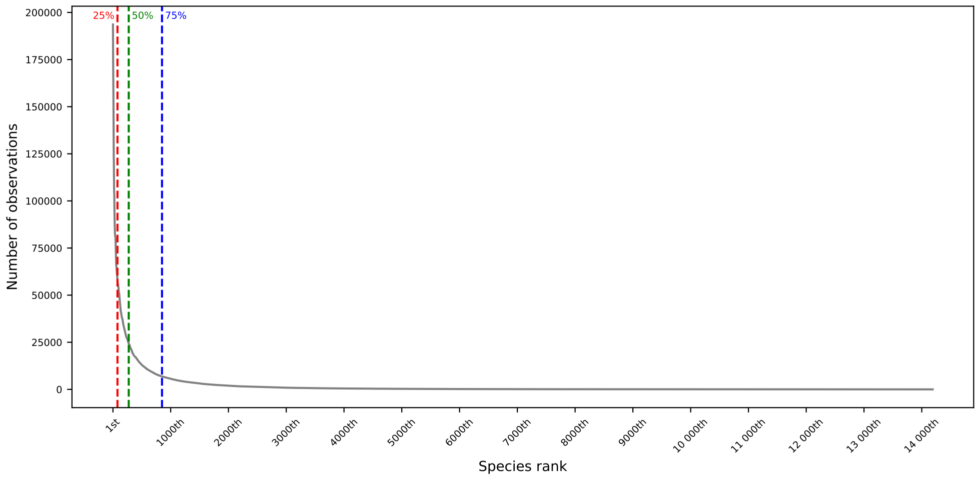

Figure 8. Long tail distribution of the whole dataset. The dashed lines represent the number of species required to obtain 25%, 50% and 75% of the total number of observations.

S15 - Syntaxon

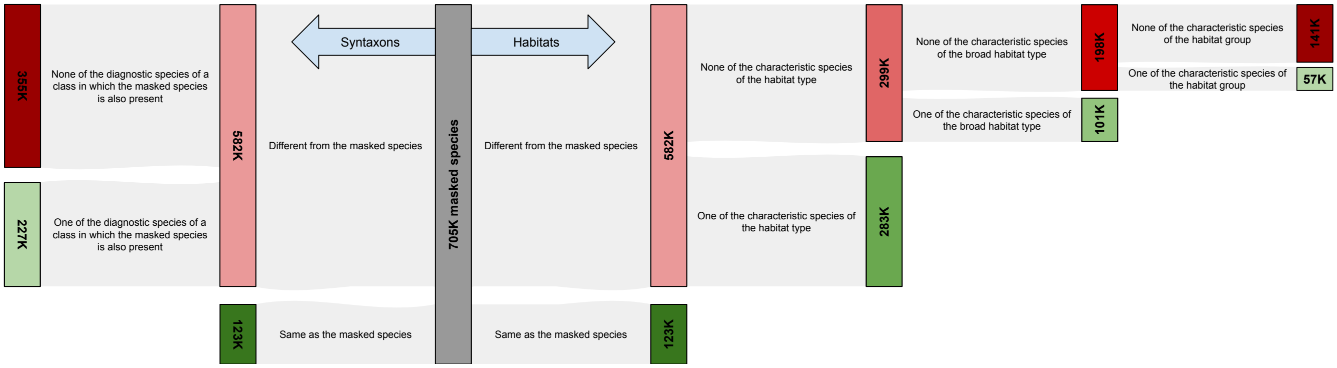

Figure 9. Results (averaged over the ten cross-validation folds) obtained by Pl@ntBERT-large-species after randomly masking a species for every vegetation plot containing ten species or more. On the right of the grey bar, we check if the predicted species is characteristic of the habitat. On the left, we check if the predicted species is diagnostic to the vegetation unit.

S16 - Hierarchy

Table 5. Details on the taxonomic rank of the 14 189 species observed in the dataset

Taxonomic rank	# Groups	Name of biggest group	# Species	Name of smallest group	# Species
Kingdom	5	Plantae	13,555	Animalia	1
Phylum	12	Tracheophyta	12,546	Chordata	1
Class	30	Magnoliopsida	10,301	Squamata	1
Order	137	Asterales	1,990	+	1
Family	400	Asteraceae	1,807	+	1
Genus	2,178	Centaurea	220	+	1

S17 - Ablation

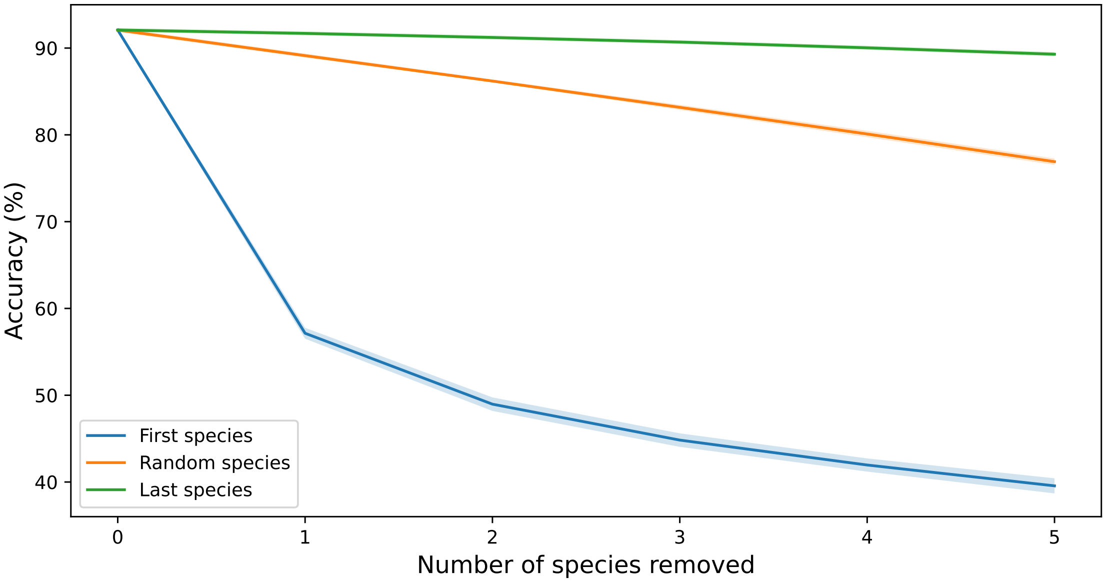

Figure 10. Accuracy (micro-averaged over the ten cross-validation folds) obtained by the base-species model after removing between 1-5 species. Only the labeled vegetation plots for which over ten species were recorded were kept in this experiment (705,479 samples out of the 850,933 samples of the text classification dataset).

S18 - Tokenization

Table 6. Comparison of the tokenization process done by several models on a list of genus names or species epithets. A ✓ indicates that the term appears in the corresponding vocabulary. Otherwise, the name is broken down into word pieces as indicated by the hyphens. These word pieces often have no botanical relevance (e.g., "g", "is", or "up") and may hinder fine-tuning for downstream tasks. Pl@ntBERT refers to the term-based version of the proposed language model. Indeed, the tokenizer of the species-based version was built such that each token is an entire species and thus no individual genus nor epithet is included in its vocabulary. On the contrary, the tokenizer of the term-based version was built such that each genus name and species epithet are included in the vocabulary. BERT and SciBERT were built such that their vocabularies contain the most frequently used words or subword units from the dataset they were trained on (Wikipedia and BookCorpus for BERT and S2ORC for SciBERT).

Botanical term	Category	BERT	SciBERT	Pl@ntBERT
Florida	Epithet	✓	✓	✓
Cannabis	Genus	✓	✓	✓
Media	Epithet	✓	✓	✓
Thymus	Genus	thy-mus	✓	✓
Glycine	Genus	g-ly-cine	✓	✓
Vulgaris	Epithet	vulgar-is	✓	✓
Paracynoglossum	Genus	para-cy-no-gl-oss-um	para-cy-no-gl-oss-um	✓
Verticillatoinundata	Epithet	ve-rti-ci-lla-to-in-unda-ta	ver-tic-illa-to-in-und-ata	✓
Contortuplicatus	Epithet	con-tort-up-lica-tus	cont-ort-up-lica-tu-s	✓

S19 - Demo

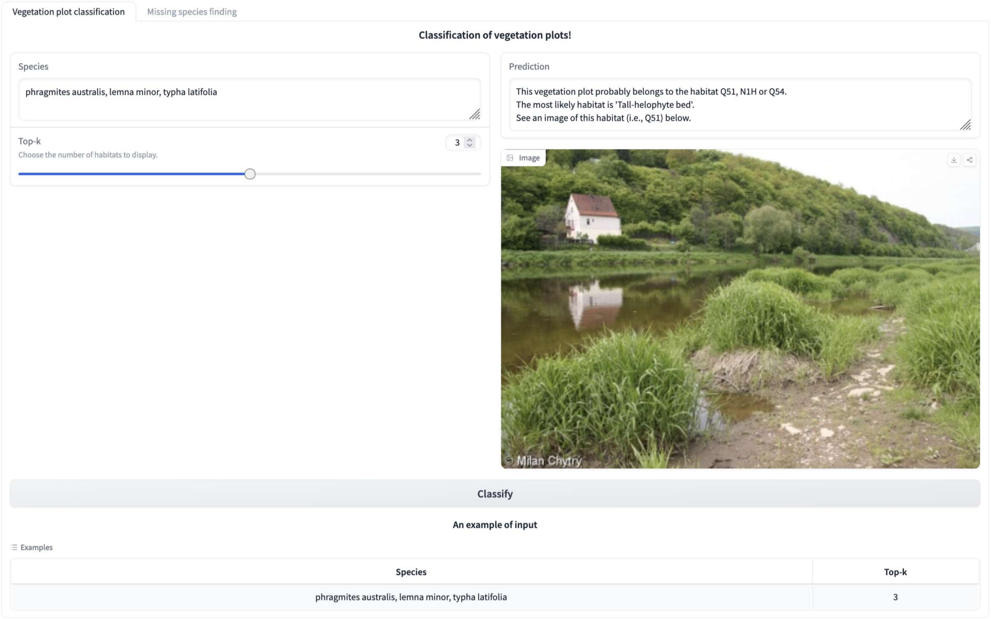

(a) Demo for the text classification task. The model accurately classifies the sentence "*phragmites australis*, *lemna minor*, *typha latifolia*" to the habitat type Q51 (*Tall-helophyte bed*).

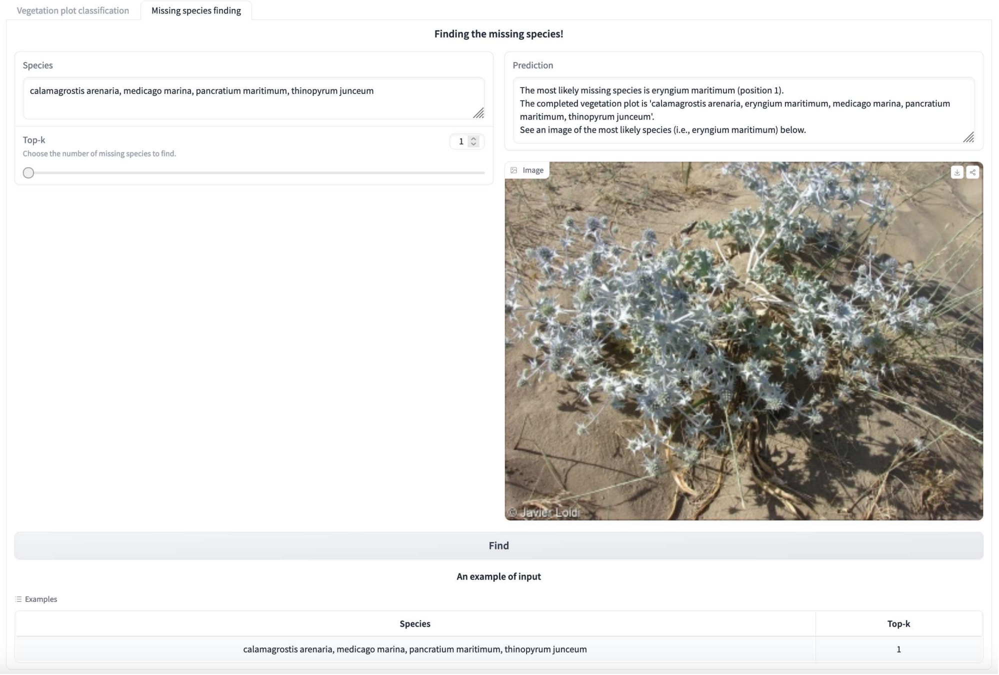

(b) Demo for the fill-mask task. The model accurately finds that the missing species in the sentence "*calamagrostis arenaria*, *medicago marina*, *pancratium maritimum*, *thinopyrum junceum*" is *eryngium maritimum* (located at the 2nd position).

Figure 11. Examples of classification (Figure 11a) and masking (Figure 11b) using the free demo. The large-species model trained on folds 1-9 is used, and the examples shown here are coming from the first fold. The images were provided by the online portal `FloraVeg.EU` and were taken by Milan Chytrý (Figure 11a) and Javier Loidi (Figure 11b). This demo of Pl@ntBERT is available here: `https://huggingface.co/spaces/CesarLeblanc/plantbert_space`.

S20 - Framework

For more detailed instructions, please refer to the official GitHub repository.

▶ Installation

Before proceeding, please make sure the following packages are installed on your system:

- Python $\geq$ 3.8
- pip
- Git
- CUDA
- Git LFS

The most straightforward way to install Pl@ntBERT is to do so via repository cloning:

```
git clone https://github.com/cesar-leblanc/plantbert.git Pl@ntBERT
cd Pl@ntBERT
```

Then, make sure that the dependencies listed in the **environment.yml** and **requirements.txt** files are installed. One way to do so is to use venv:

```
python -m venv ~/environments/pl@ntbert
source ~/environments/pl@ntbert/bin/activate
pip install -r requirements.txt
```

Make sure you installed the pre-trained and fine-tuned models:

```
git lfs install
git clone https://huggingface.co/CesarLeblanc/bert-base-uncased
Models/bert-base-uncased
git clone https://huggingface.co/CesarLeblanc/bert-large-uncased
Models/bert-large-uncased
git clone https://huggingface.co/CesarLeblanc/plantbert_fill_mask_model
Models/plantbert_fill_mask_model
git clone https://huggingface.co/CesarLeblanc/plantbert_text_classification_model
Models/plantbert_text_classification_model
```

Starting from this point, all commands have to be launched within the **Scripts** folder:

```
cd Scripts
```

Check that the installation went well by using the following command:

```
python main.py --pipeline check
```

▶ Dataset

To cure a dataset, two comma-separated values files (e.g., from the European Vegetation Archive) should be placed in the folder **Pl@ntBERT/Data/**:

- **header.csv**: the header file, linked to the species file by the plot identifiers, which contains plot attributes. Four columns are required:
  1. PlotObservationID (integers): vegetation plot identifier
  2. Habitat (strings): vegetation plot habitat type (EUNIS)
  3. Longitude (floats): vegetation plot longitude (WGS 84 decimal degrees)
  4. Latitude (floats): vegetation plot latitude (WGS 84 decimal degrees)

- **species.csv**: the species file, linked to the header file by the plot identifiers, which contains vegetation-plot data. Three columns are required:
  1. PlotObservationID (integers): vegetation plot identifier
  2. Species (strings): species name
  3. Cover (floats): species percentage cover

Here is the most straightforward way to cure a dataset for Pl@ntBERT:

```
python main.py --pipeline curation
```

► Training

Having two cured vegetation datasets (i.e., the unlabeled **plantbert_fill_mask_dataset** that only contains list of co-occurring species and the labeled **plantbert_text_classification_dataset** that contains list of co-occurring species along with the associated habitat types in the folder **Pl@ntBERT/Datasets/**) is necessary to train models. Here is the most straightforward way to train a Pl@ntBERT model:

```
python main.py --pipeline masking classification
```

► Inference

After training a model (i.e., a model whose name matches your choice of parameters should be present in the folder **Pl@ntBERT/Models/**), you can use it to identify habitat types and find missing species of new vegetation plots by placing into the folder **Pl@ntBERT/Datasets/** a comma-separated value file named **vegetation_plots.csv** containing only one column:

- Observations (strings): a list of comma-separated names of species, ranked (if possible) in order of abundance

Here is the most straightforward way to run inference using Pl@ntBERT:

```
python main.py --pipeline inference
```

Two new columns named "Habitat" and "Species" will be added to the file, containing for each line (i.e., for each vegetation plot) the EUNIS code of the most likely habitat type and the scientific name of the most likely missing species.

S21 - Parameters

Table 7. Parameters of the neural network used for missing species retrieval

Parameter	Description	Value
hidden_layer_size	Number of neurons in the hidden layer	100
activation	Activation function for the hidden layer	relu
solver	Solver for weight optimization	adam
alpha	Strength of the L2 regularization term	0.0001
batch_size	Size of minibatches for stochastic optimizers	200
learning_rate	Learning rate schedule for weight updates	constant
learning_rate_init	The initial learning rate used	0.001
max_iter	Maximum number of iterations	200
shuffle	Whether to shuffle samples in each iteration	True
random_state	Determines random number generation	123
tol	Tolerance for the optimization	1e-4
early_stopping	Whether to terminate training when validation score is not improving	False
beta_1	Exponential decay rate for estimates of first moment vector	0.9
beta_2	Exponential decay rate for estimates of second moment vector	0.999
epsilon	Value for numerical stability	1e-8
n_iter_no_change	Maximum number of epochs to not meet tol improvement	10

S22 - Performance

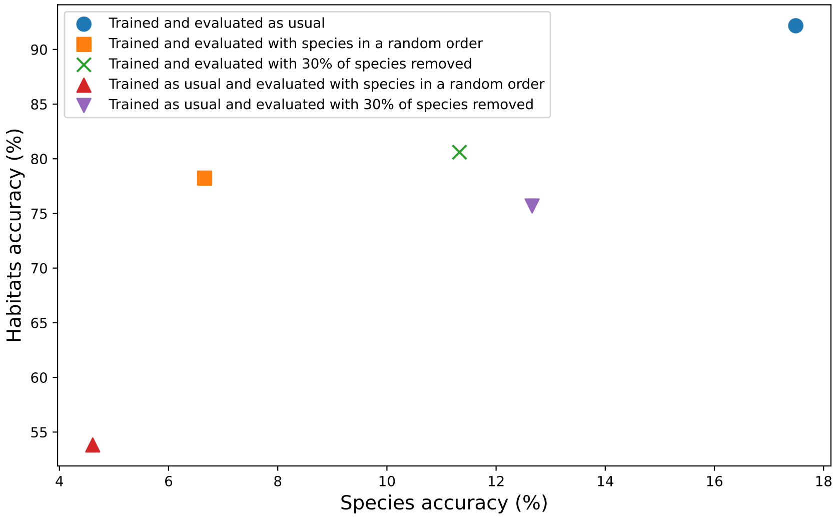

Figure 12. Masking and classification accuracies (micro-averaged over the ten cross-validation folds) obtained by the large-species model using different training and evaluation settings. For the fill-mask task, exactly one species from each vegetation plot was masked (randomly), and the first prediction of the model that was not a species already present in the sentence was kept. Only the labeled vegetation plots for which over ten species were recorded were kept in the test set (for the dropout setting, we kept the vegetation plots that still contained over ten species after the removal of random species).

S23 - Examples

Text classification examples

Sentence 1: *phragmites australis*, *lemna minor*, *typha latifolia*
Label 1: *Q51*

Sentence 2: *erica arborea*, *erica scoparia*, *quercus ilex*, *calluna vulgaris*, *castanea sativa*, *phillyrea angustifolia*, *pinus pinaster*, *robinia pseudoacacia*, *rubia peregrina*, *teucrium scorodonia*, *crataegus monogyna*, *cytisus scoparius*, *erica cinerea*, *genista pilosa*, *poa nemoralis*
Label 2: *T21*

Fill-mask examples

Sentence 1: *calamagrostis arenaria*, [MASK], *medicago marina*, *pancratium maritimum*, *thinopyrum junceum*
Label 1: *eryngium maritimum*

Sentence 2: *quercus cerris*, *carpinus orientalis*, [MASK], *primula vulgaris*, *brachypodium sylvaticum*, *campanula rapunculus*, *carpinus betulus*, *cornus mas*, *fraxinus ornus*, *galium sylvaticum*, [MASK], *knautia drymeia*, *lathyrus niger*, *luzula forsteri*, *pteridium aquilinum*, *viola alba*
Label 2: *helleborus odorus* and *hedera helix*

S24 - Dataset

Table 8. Overview of the EUNIS habitat types and the number of vegetation plots assigned to each of them, with the corresponding EUNIS codes and European Red List of Habitats categories. The numbers of vegetation plots for the broad habitat groups and habitat groups (respectively the levels 1 and 2 of the classification hierarchy, in bold for broad habitat groups and both denoted by gray rows) are the sums of the numbers of vegetation plots for the subordinated habitat types. The European Red List of Habitats categories for the broad habitat groups and habitat groups are the biggest risks of collapse of the categories of vegetation plots for the subordinated habitat types. The total number of vegetation plots in the text-classification dataset is 850 933.

Begin of Table 8

EUNIS code	EUNIS name	Red List category	Number of vegetation plots
**MA2**	**Littoral biogenic habitats**	**Endangered**	**28,952**
MA21	Arctic littoral biogenic habitats	Near Threatened	340
MA211	Arctic coastal saltmarsh	Near Threatened	340
MA22	Atlantic littoral biogenic habitats	Vulnerable	19,854
MA221	Atlantic saltmarsh driftline	Vulnerable	119
MA222	Atlantic upper saltmarsh	Vulnerable	873
MA223	Atlantic upper-mid saltmarsh and saline and brackish reed, rush and sedge bed	Vulnerable	7,316
MA224	Atlantic mid-low saltmarsh	Vulnerable	10,377
MA225	Atlantic pioneer saltmarsh	Vulnerable	1,169
MA23	Baltic hydrolittoral biogenic habitats	Endangered	760
MA232	Baltic coastal meadow	Endangered	760
MA24	Black Sea littoral biogenic habitats	Near Threatened	2,956
MA241	Black Sea littoral saltmarsh	Near Threatened	2,956
MA25	Mediterranean littoral biogenic habitats	Near Threatened	5,042
MA251	Mediterranean upper saltmarsh	Near Threatened	406
MA252	Mediterranean upper-mid saltmarsh and saline and brackish reed, rush and sedge bed	Near Threatened	1,666
MA253	Mediterranean mid-low saltmarsh	Near Threatened	2,970
**N**	**Coastal habitats**	**Endangered**	**33,121**
N1	Coastal dunes and sandy shores	Endangered	29,197
N11	Atlantic, Baltic and Arctic sand beach	Vulnerable	566
N12	Mediterranean and Black Sea sand beach	Near Threatened	1,725
N13	Atlantic and Baltic shifting coastal dune	Near Threatened	3,465
N14	Mediterranean, Macaronesian and Black Sea shifting coastal dune	Vulnerable	6,102
N15	Atlantic and Baltic coastal dune grassland (grey dune)	Vulnerable	2,770
N16	Mediterranean and Macaronesian coastal dune grassland (grey dune)	Endangered	2,886
N17	Black Sea coastal dune grassland (grey dune)	Endangered	735
N18	Atlantic and Baltic coastal Empetrum heath	Vulnerable	325
N19	Atlantic coastal Calluna and Ulex heath	Least Concern	226
N1A	Atlantic and Baltic coastal dune scrub	Least Concern	3,207
N1B	Mediterranean and Black Sea coastal dune scrub	Vulnerable	445
N1C	Macaronesian coastal dune scrub	Endangered	67
N1D	Atlantic and Baltic broad-leaved coastal dune forest	Least Concern	965
N1E	Black Sea broad-leaved coastal dune forest	Endangered	21
N1F	Baltic coniferous coastal dune forest	Vulnerable	480
N1G	Mediterranean coniferous coastal dune forest	Least Concern	227
N1H	Atlantic and Baltic moist and wet dune slack	Vulnerable	4,517

Continuation of Table 8			
EUNIS code	EUNIS name	Red List category	Number of vegetation plots
N1J	Mediterranean and Black Sea moist and wet dune slack	Least Concern	468
N2	Coastal shingle	Least Concern	696
N21	Atlantic, Baltic and Arctic coastal shingle beach	Least Concern	600
N22	Mediterranean and Black Sea coastal shingle beach	Least Concern	96
N3	Rock cliffs, ledges and shores, including the supralittoral	Least Concern	3,228
N31	Atlantic and Baltic rocky sea cliff and shore	Least Concern	166
N32	Mediterranean and Black Sea rocky sea cliff and shore	Least Concern	2,823
N33	Macaronesian rocky sea cliff and shore	Least Concern	54
N34	Atlantic and Baltic soft sea cliff	Least Concern	62
N35	Mediterranean and Black Sea soft sea cliff	Data Deficient	123
**Q**	**Wetlands**	**Data Deficient**	**92,493**
Q1	Raised and blanket bogs	Data Deficient	5,080
Q11	Raised bog	Data Deficient	4,009
Q12	Blanket bog	Data Deficient	1,071
Q2	Valley mires, poor fens and transition mires	Data Deficient	19,985
Q21	Oceanic valley mire	Data Deficient	2,081
Q22	Poor fen	Data Deficient	6,071
Q23	Relict mire of Mediterranean mountains	Data Deficient	113
Q24	Intermediate fen and soft-water spring mire	Data Deficient	5,722
Q25	Non-calcareous quaking mire	Data Deficient	5,998
Q4	Base-rich fens and calcareous spring mires	Data Deficient	9,096
Q41	Alkaline, calcareous, carbonate-rich small-sedge spring fen	Data Deficient	2,986
Q42	Extremely rich moss-sedge fen	Data Deficient	2,411
Q43	Tall-sedge base-rich fen	Data Deficient	1,188
Q44	Calcareous quaking mire	Data Deficient	1,395
Q45	Arctic-alpine rich fen	Data Deficient	1,101
Q46	Carpathian travertine fen with halophytes	Data Deficient	15
Q5	Helophyte beds	Data Deficient	58,332
Q51	Tall-helophyte bed	Data Deficient	31,635
Q52	Small-helophyte bed	Data Deficient	14,487
Q53	Tall-sedge bed	Data Deficient	9,298
Q54	Inland saline or brackish helophyte bed	Data Deficient	2,912
**R**	**Grasslands and lands dominated by forbs, mosses or lichens**	**Critically Endangered**	**291,567**
R1	Dry grasslands	Critically Endangered	85,761
R11	Pannonian and Pontic sandy steppe	Critically Endangered	393
R12	Cryptogam- and annual-dominated vegetation on siliceous rock outcrops	Vulnerable	596
R13	Cryptogam- and annual-dominated vegetation on calcareous and ultramafic rock outcrops	Vulnerable	2,586
R14	Perennial rocky grassland of the Italian Peninsula	Vulnerable	968
R15	Continental dry rocky steppic grassland and dwarf scrub on chalk outcrops	Data Deficient	489
R16	Perennial rocky grassland of Central and South-Eastern Europe	Least Concern	6,582
R17	Heavy-metal dry grassland of the Balkans	Least Concern	69

Continuation of Table 8			
EUNIS code	EUNIS name	Red List category	Number of vegetation plots
R18	Perennial rocky calcareous grassland of subatlantic-submediterranean Europe	Vulnerable	4,359
R19	Dry steppic submediterranean pasture of the Amphi-Adriatic region	Near Threatened	677
R1A	Semi-dry perennial calcareous grassland (meadow steppe)	Vulnerable	42,206
R1B	Continental dry grassland (true steppe)	Near Threatened	8,237
R1C	Desert steppe	Data Deficient	617
R1D	Mediterranean closely grazed dry grassland	Least Concern	1,087
R1E	Mediterranean tall perennial dry grassland	Least Concern	1,806
R1F	Mediterranean annual-rich dry grassland	Near Threatened	1,465
R1G	Iberian oromediterranean siliceous dry grassland	Near Threatened	238
R1H	Iberian oromediterranean basiphilous dry grassland	Least Concern	282
R1J	Cyrno-Sardean oromediterranean siliceous dry grassland	Endangered	41
R1K	Balkan and Anatolian oromediterranean dry grassland	Least Concern	89
R1M	Lowland to montane, dry to mesic grassland usually dominated by Nardus stricta	Vulnerable	2,907
R1P	Oceanic to subcontinental inland sand grassland on dry acid and neutral soils	Endangered	5,364
R1Q	Inland sanddrift and dune with siliceous grassland	Endangered	2,041
R1R	Mediterranean to Atlantic open, dry, acid and neutral grassland	Near Threatened	2,555
R1S	Heavy-metal grassland in Western and Central Europe	Endangered	107
R2	Mesic grasslands	Vulnerable	100,615
R21	Mesic permanent pasture of lowlands and mountains	Vulnerable	29,849
R22	Low and medium altitude hay meadow	Vulnerable	69,533
R23	Mountain hay meadow	Vulnerable	1,202
R24	Iberian summer pasture (vallicar)	Near Threatened	31
R3	Seasonally wet and wet grasslands	Endangered	56,414
R31	Mediterranean tall humid inland grassland	Least Concern	1,765
R32	Mediterranean short moist grassland of lowlands	Least Concern	175
R33	Mediterranean short moist grassland of mountains	Least Concern	691
R34	Submediterranean moist meadow	Least Concern	871
R35	Moist or wet mesotrophic to eutrophic hay meadow	Endangered	27,392
R36	Moist or wet mesotrophic to eutrophic pasture	Endangered	15,609
R37	Temperate and boreal moist or wet oligotrophic grassland	Endangered	9,911
R4	Alpine and subalpine grasslands	Vulnerable	16,892
R41	Snow-bed vegetation	Vulnerable	1,059
R42	Boreal and Arctic acidophilous alpine grassland	Least Concern	462
R43	Temperate acidophilous alpine grassland	Least Concern	8,713
R44	Arctic-alpine calcareous grassland	Least Concern	5,921
R45	Alpine and subalpine calcareous grassland of the Balkans and Apennines	Least Concern	737

Continuation of Table 8

EUNIS code	EUNIS name	Red List category	Number of vegetation plots
R5	Woodland fringes and clearings and tall forb stands	Vulnerable	26,422
R51	Thermophilous forest fringe of base-rich soils	Near Threatened	669
R52	Forest fringe of acidic nutrient-poor soils	Least Concern	443
R53	Macaronesian thermophilous forest fringe	Near Threatened	30
R54	Pteridium aquilinum vegetation	Least Concern	1,331
R55	Lowland moist or wet tall-herb and fern fringe	Vulnerable	18,844
R56	Montane to subalpine moist or wet tall-herb and fern fringe	Least Concern	2,852
R57	Herbaceous forest clearing vegetation	Data Deficient	2,253
R6	Inland salt steppes	Endangered	5,463
R61	Mediterranean inland salt steppe	Vulnerable	480
R62	Continental inland salt steppe	Vulnerable	2,198
R63	Temperate inland salt marsh	Endangered	2,520
R64	Semi-desert salt pan	Data Deficient	191
R65	Continental subsaline alluvial pasture and meadow	Data Deficient	74
**S**	**Heathlands, scrub and tundra**	**Endangered**	**61,325**
S1	Tundra	Least Concern	1,156
S11	Shrub tundra	Least Concern	1,108
S12	Moss and lichen tundra	Least Concern	48
S2	Arctic, alpine and subalpine scrub	Least Concern	14,611
S21	Subarctic and alpine dwarf Salix scrub	Least Concern	2,082
S22	Alpine and subalpine ericoid heath	Least Concern	7,163
S23	Alpine and subalpine Juniperus scrub	Least Concern	1,937
S24	Subalpine genistoid scrub of the Amphi-Adriatic region	Least Concern	138
S25	Subalpine and subarctic deciduous scrub	Least Concern	1,115
S26	Subalpine Pinus mugo scrub	Least Concern	2,176
S3	Temperate and Mediterranean-montane scrub	Vulnerable	12,693
S31	Lowland to montane temperate and submediterranean Juniperus scrub	Least Concern	976
S32	Temperate Rubus scrub	Data Deficient	2,007
S33	Lowland to montane temperate and submediterranean genistoid scrub	Least Concern	1,821
S34	Balkan-Anatolian submontane genistoid scrub	Vulnerable	162
S35	Temperate and submediterranean thorn scrub	Least Concern	5,072
S36	Low steppic scrub	Least Concern	694
S37	Corylus avellana scrub	Least Concern	1,358
S38	Temperate forest clearing scrub	Data Deficient	603
S4	Temperate heathland	Vulnerable	9,649
S41	Wet heath	Vulnerable	2,841
S42	Dry heath	Vulnerable	6,808
S5	Maquis, arborescent matorral and thermo-Mediterranean scrub	Vulnerable	6,034
S51	Mediterranean maquis and arborescent matorral	Least Concern	4,353
S52	Submediterranean pseudomaquis	Least Concern	781
S53	Spartium junceum scrub	Data Deficient	427
S54	Thermomediterranean arid scrub	Vulnerable	473
S6	Garrigue	Least Concern	5,837
S61	Western basiphilous garrigue	Least Concern	3,610
S62	Western acidophilous garrigue	Least Concern	200

Continuation of Table 8			
EUNIS code	EUNIS name	Red List category	Number of vegetation plots
S63	Eastern garrigue	Least Concern	642
S64	Macaronesian garrigue	Data Deficient	87
S65	Mediterranean gypsum scrub	Least Concern	830
S66	Mediterranean halo-nitrophilous scrub	Least Concern	201
S67	Aralo-Caspian semi-desert	Data Deficient	222
S68	Semi-desert sand dune with sparse scrub	Data Deficient	45
S7	Spiny Mediterranean heaths	Least Concern	2,839
S71	Western Mediterranean spiny heath	Least Concern	137
S72	Eastern Mediterranean spiny heath (phrygana)	Least Concern	606
S73	Western Mediterranean mountain hedgehog-heath	Least Concern	424
S74	Central Mediterranean mountain hedgehog-heath	Least Concern	603
S75	Eastern Mediterranean mountain hedgehog-heath	Least Concern	871
S76	Canarian mountain hedgehog-heath	Least Concern	198
S8	Thermo-Atlantic xerophytic scrub	Endangered	404
S81	Canarian xerophytic scrub	Vulnerable	384
S82	Madeiran xerophytic scrub	Endangered	20
S9	Riverine and fen scrub	Near Threatened	8102
S91	Temperate riparian scrub	Least Concern	2,630
S92	Salix fen scrub	Near Threatened	4,590
S93	Mediterranean riparian scrub	Least Concern	813
S94	Semi-desert riparian scrub	Data Deficient	69
**T**	**Forests and other wooded land**	**Endangered**	**241,834**
T1	Broadleaved deciduous forests	Endangered	156,722
T11	Temperate Salix and Populus riparian forest	Near Threatened	2,832
T12	Alnus glutinosa-Alnus incana forest on riparian and mineral soils	Least Concern	10,578
T13	Temperate hardwood riparian forest	Endangered	10,083
T14	Mediterranean and Macaronesian riparian forest	Vulnerable	1,036
T15	Broadleaved swamp forest on non-acid peat	Vulnerable	2,980
T16	Broadleaved mire forest on acid peat	Vulnerable	3,753
T17	Fagus forest on non-acid soils	Near Threatened	39,539
T18	Fagus forest on acid soils	Near Threatened	9,210
T19	Temperate and submediterranean thermophilous deciduous forest	Least Concern	21,260
T1A	Mediterranean thermophilous deciduous forest	Least Concern	580
T1B	Acidophilous Quercus forest	Vulnerable	11,721
T1C	Temperate and boreal mountain Betula and Populus tremula forest on mineral soils	Least Concern	292
T1D	Southern European mountain Betula and Populus tremula forest on mineral soils	Least Concern	27
T1E	Carpinus and Quercus mesic deciduous forest	Near Threatened	29,573
T1F	Ravine forest	Least Concern	8,287
T1G	Alnus cordata forest	Data Deficient	112
T1H	Broadleaved deciduous plantation of non site-native trees	Data Deficient	4,859
T2	Broadleaved evergreen forests	Vulnerable	14,610
T21	Mediterranean evergreen Quercus forest	Least Concern	11,846
T22	Mainland laurophyllous forest	Least Concern	268
T23	Macaronesian laurophyllous forest	Vulnerable	85
T24	Olea europaea-Ceratonia siliqua forest	Least Concern	1,589
T25	Phoenix theophrasti vegetation	Least Concern	27

Continuation of Table 8			
EUNIS code	EUNIS name	Red List category	Number of vegetation plots
T27	Ilex aquifolium forest	Least Concern	712
T28	Macaronesian heathy forest	Vulnerable	52
T29	Broadleaved evergreen plantation of non site-native trees	Data Deficient	31
T3	Coniferous forests	Vulnerable	70,502
T31	Temperate mountain Picea forest	Least Concern	13,346
T32	Temperate mountain Abies forest	Least Concern	9,616
T33	Mediterranean mountain Abies forest	Least Concern	586
T34	Temperate subalpine Larix, Pinus cembra and Pinus uncinata forest	Near Threatened	2,056
T35	Temperate continental Pinus sylvestris forest	Near Threatened	9,694
T36	Temperate and submediterranean montane Pinus sylvestris-Pinus nigra forest	Least Concern	2,267
T37	Mediterranean montane Pinus sylvestris-Pinus nigra forest	Least Concern	874
T38	Mediterranean montane Cedrus forest	Vulnerable	594
T39	Mediterranean and Balkan subalpine Pinus heldreichii-Pinus peuce forest	Least Concern	398
T3A	Mediterranean lowland to submontane Pinus forest	Least Concern	6,811
T3B	Pinus canariensis forest	Least Concern	659
T3C	Taxus baccata forest	Least Concern	252
T3D	Mediterranean Cupressaceae forest	Least Concern	975
T3E	Macaronesian Juniperus forest	Vulnerable	20
T3F	Dark taiga	Near Threatened	3,530
T3G	Pinus sylvestris light taiga	Least Concern	3,505
T3H	Larix light taiga	Data Deficient	34
T3J	Pinus and Larix mire forest	Data Deficient	4,000
T3K	Picea mire forest	Data Deficient	2,091
T3M	Coniferous plantation of non site-native trees	Data Deficient	9,194
**U**	**Inland habitats with no or little soil and mostly with sparse vegetation**	**Least Concern**	**7,806**
U2	Screes	Least Concern	3,906
U21	Boreal and arctic siliceous scree and block field	Least Concern	30
U22	Temperate high-mountain siliceous scree	Least Concern	788
U23	Temperate, lowland to montane siliceous scree	Least Concern	132
U24	Mediterranean siliceous scree	Data Deficient	127
U25	Boreal and arctic base-rich scree and block field	Data Deficient	38
U26	Temperate high-mountain base-rich scree and moraine	Least Concern	1,161
U27	Temperate, lowland to montane base-rich scree	Least Concern	1,227
U28	Western Mediterranean base-rich scree	Least Concern	183
U29	Eastern Mediterranean base-rich scree	Least Concern	205
U2A	Crimean base-rich scree	Data Deficient	15
U3	Inland cliffs, rock pavements and outcrops	Least Concern	3,823
U32	Temperate high-mountain siliceous inland cliff	Least Concern	182
U33	Temperate, lowland to montane siliceous inland cliff	Least Concern	301
U34	Mediterranean siliceous inland cliff	Least Concern	182
U35	Boreal and arctic base-rich inland cliff	Data Deficient	12
U36	Temperate high-mountain base-rich inland cliff	Least Concern	657

Continuation of Table 8			
EUNIS code	EUNIS name	Red List category	Number of vegetation plots
U37	Temperate, lowland to montane base-rich inland cliff	Least Concern	1,541
U38	Mediterranean base-rich inland cliff	Least Concern	736
U3A	Temperate ultramafic inland cliff	Data Deficient	49
U3B	Mediterranean ultramafic inland cliff	Data Deficient	39
U3C	Macaronesian inland cliff	Least Concern	38
U3D	Wet inland cliff	Data Deficient	86
U6	Recent volcanic features	Least Concern	77
U61	Subarctic volcanic field	Least Concern	20
U62	Mediterranean, Macaronesian and temperate volcanic field	Least Concern	57
**V**	**Vegetated man-made habitats**	**Endangered**	**93,835**
V1	Arable land and market gardens	Endangered	40,768
V11	Intensive unmixed crops	Data Deficient	7,662
V12	Mixed crops of market gardens and horticulture	Data Deficient	446
V13	Arable land with unmixed crops grown by low-intensity agricultural methods	Endangered	3,505
V14	Inundated or inundatable cropland, including rice fields	Data Deficient	216
V15	Bare tilled, fallow or recently abandoned arable land	Data Deficient	28,939
V3	Artificial grasslands and herb-dominated habitats	Data Deficient	53,067
V32	Mediterranean subnitrophilous annual grasslands	Data Deficient	10,534
V33	Dry mediterranean lands with unpalatable non-vernal herbaceous vegetation	Data Deficient	559
V34	Trampled xeric grassland with annuals	Data Deficient	2,489
V35	Trampled mesophilous grassland with annuals	Data Deficient	4,497
V37	Annual anthropogenic herbaceous vegetation	Data Deficient	14,143
V38	Dry perennial anthropogenic herbaceous vegetation	Data Deficient	16,684
V39	Mesic perennial anthropogenic herbaceous vegetation	Data Deficient	4,161
End of Table 8			

S25 - Co-occurrence

The formula used to predict missing species based on the co-occurrence matrix is:

$$P(s_j \mid \text{species list}) = \frac{\prod_{s_i \in \text{species list}} P(s_i|s_j) \times P(s_j)}{P(\text{species list})} \propto \prod_{s_i \in \text{species list}} P(s_i|s_j) \times P(s_j)$$

where:

- $P(s_j \mid \text{species list})$ is the probability of species s_j co-occurring with the given species list.
- $P(s_i \mid s_j)$ is computed as the co-occurrence $C(s_i, s_j)$ between species s_i and s_j.
- $P(s_j)$ is computed as $F(s_j)$, the frequency of species s_j, taken from the diagonal of the matrix.

Then the prediction solves:

$$\hat{s} = \underset{s \notin \text{species list}}{\arg\max} \; p(s \mid \text{species list})$$

S26 - Workflow

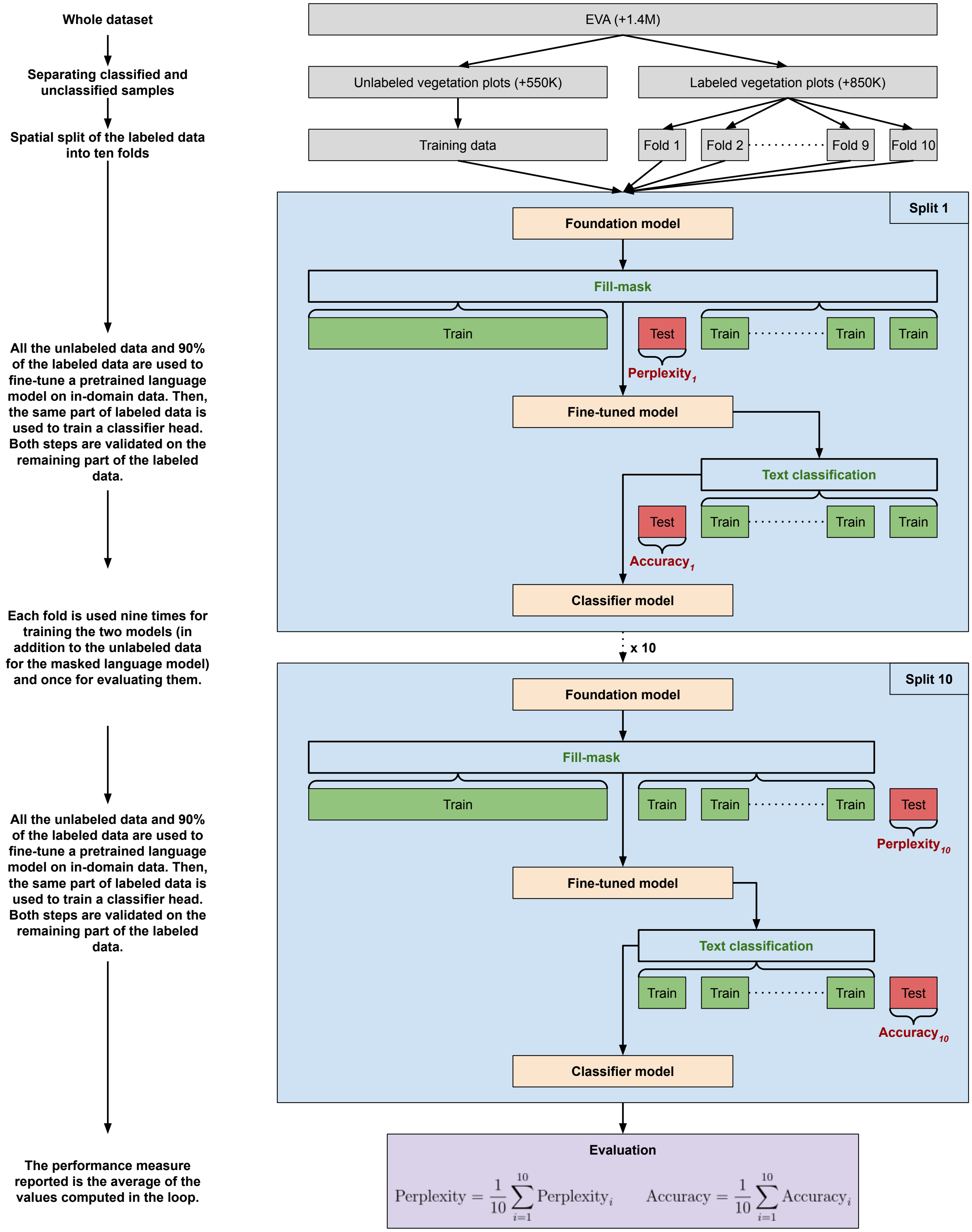

Figure 13. Overview of the 10-fold cross-validation approach used in this paper.

S27 - Categories

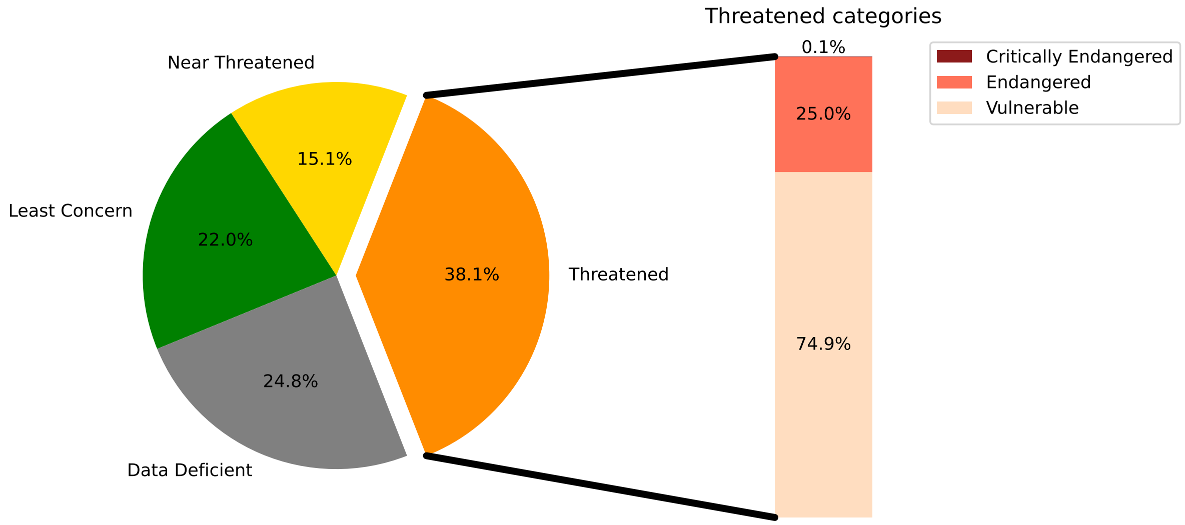

Figure 14. Distribution of the European Red List of Habitats categories across the 850,933 labeled vegetation plots of the text classification dataset.

S28 - Explainability

[CLS] calamagrostis arenaria , achillea maritima , calystegia soldanella , cyperus capitatus , eryngium maritimum , medicago marina , thinopyrum junceum [SEP]

(a) Sentence visualization showing how positively (green highlighted parts), negatively (red highlighted parts), or neutrally (white highlighted parts) each species is contributing to the classification of the vegetation plot. The saturation of the colors shows the magnitude of the contribution.

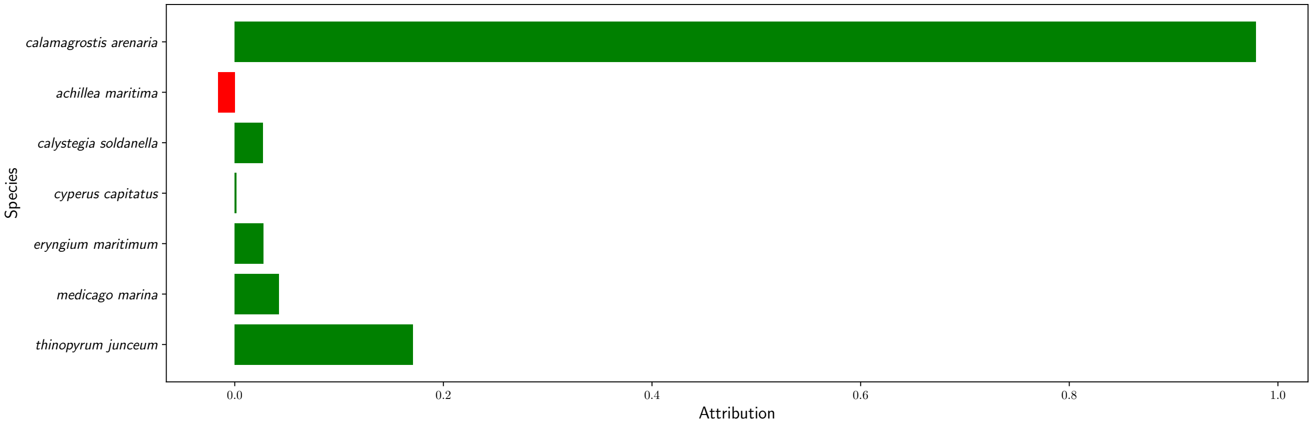

(b) Bar chart showing how each species contributed to the classification of the vegetation plot. The species with positive values (green bars going right) indicate a positive contribution, and the species with negative values (red bars going left) indicate the opposite. The length of the bars shows the magnitude of the contribution.

Figure 15. Explainability of Pl@ntBERT (large-species version trained on folds 1-9) with integrated gradients using a vegetation plot from the fold 0. The vegetation plot belongs to the habitat type *N14* and is correctly assigned to this label by the model. This habitat type has two dominant species: *Calamagrostis arenaria* and *Thinopyrum junceum*. Both subfigures show that it is mainly these two species that seem to drive the classification of the vegetation plot to the habitat type *N14*.

S29 - Patterns

Table 9. Five samples of vegetation patterns consisting of three plants *ABC*, where species *A* and *C* never appear if species *B* is missing (in the fill-mask dataset and in the folds 1-9 of the text classification dataset). Species *B* was removed, and Pl@ntBERT-large-species, the co-occurrence matrix, and the neural network tried to predict the missing species. As the assemblages are impossible without species *B*, this species is expected to be output. The number represents the number of times species *A*, *B*, and *C* co-occur (which is also the number of times species *A* and *C* co-occur).

Species A	Species B	Species C	#	Model prediction	Matrix prediction	Network prediction
Allium acutiflorum	*Brachypodium retusum*	*Ceratonia siliqua*	323	*Brachypodium retusum*	*Dactylis glomerata*	*Rhododendron hirsutum*
Allium acutiflorum	*Pistacia lentiscus*	*Myrtus communis*	279	*Pistacia lentiscus*	*Dactylis glomerata*	*Atriplex prostrata*
Calamagrostis epigejos	*Salix repens*	*Vaccinium macrocarpon*	201	*Salix repens*	*Holcus lanatus*	*Phragmites australis*
Asparagus acutifolius	*Rubia peregrina*	*Leucanthemum discoideum*	156	*Rubia peregrina*	*Dactylis glomerata*	*Phragmites australis*
Gastridium phleoides	*Lagoecia cuminoides*	*Pterocephalus plumosus*	145	*Lagoecia cuminoides*	*Dactylis glomerata*	*Potamogeton crispus*

S30 - Acronyms

AI - Artificial Intelligence
API - Application Programming Interface
BERT - Bidirectional Encoder Representations from Transformers
CV - Cross-Validation
ESy - Expert System
EUNIS - EUropean Nature Information System
EVA - European Vegetation Archive
FTT - Feature Tokenizer + Transformer
GBIF - Global Biodiversity Information Facility
HDM - Habitat Distribution Modeling
LLM - Large Language Model
MLP - Multilayer Perceptron
NLP - Natural Language Processing
RFC - Random Forest Classifier
TNC - TabNet Classifier
XGB - eXtreme Gradient Boosting
WGS - World Geodetic System

S31 - Terms

artificial intelligence - the field of computer science creating systems that can perform tasks requiring human-like intelligence
attention - a mechanism in neural networks that focuses on relevant parts of the input data for better prediction
habitat group - the habitats at the level 2 of the EUNIS hierarchy
cross-validation - a statistical method used to estimate the performance of a model by partitioning data into subsets
deep learning - the subset of machine learning methods based on neural networks with representation learning
expert system - a computer system simulating the decision-making ability of a human expert
fill-mask - the task of masking some of the words in a sentence and predicting which words should replace those masks
broad habitat groups - the habitats at the level 1 of the EUNIS hierarchy
habitat types - the habitats at the level 3 of the EUNIS hierarchy
integrated gradients - an attribution method computing the path integral of gradients from a baseline input to the actual input
machine learning - a subset of AI where models learn patterns from data to make predictions without explicit instructions
natural language processing - a field of AI focused on the interaction between computers and humans through natural language
text classification - the task of assigning a label or class to a given text
token - the smallest unit of text in natural language processing, such as a word, subword, or character
tokenizer - a tool that converts text into tokens, preparing it for processing by language models
transformer - a deep learning architecture based on the multi-head attention mechanism
vegetation classification - the task of assigning vegetation plots to established classification systems
vegetation plot - standardized record of plant community composition of a defined small area

S32 - Visualization

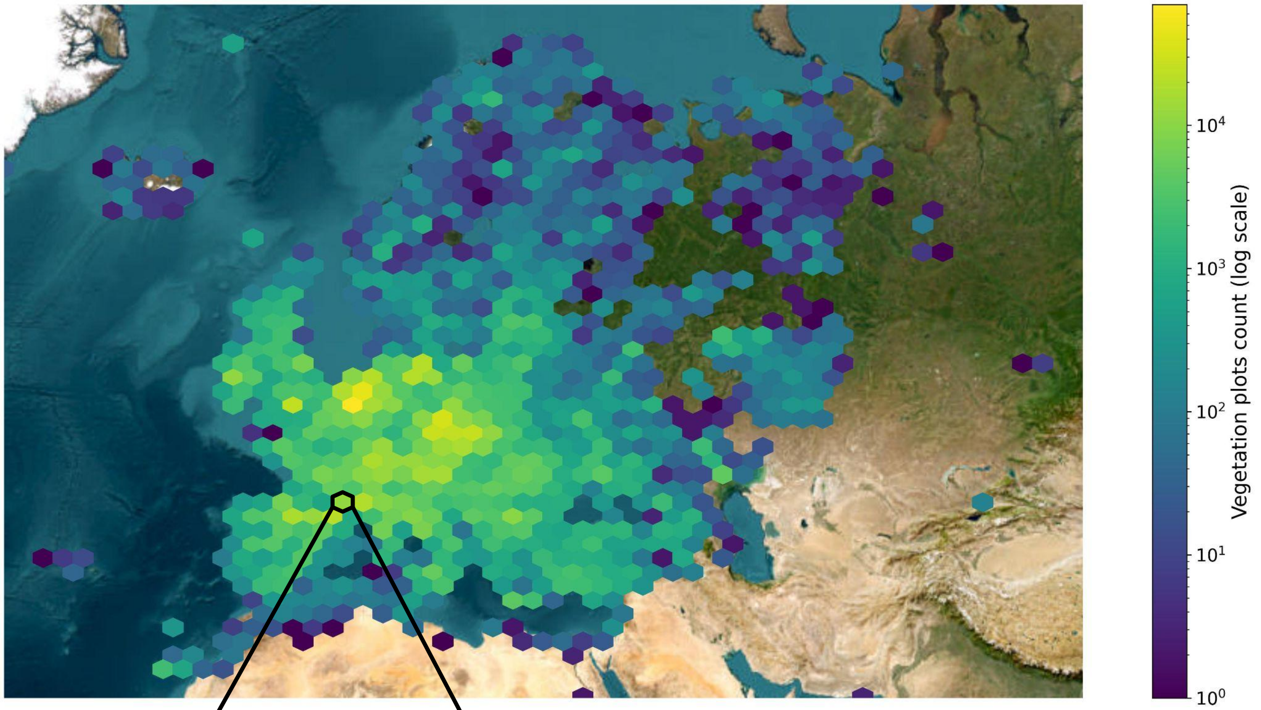

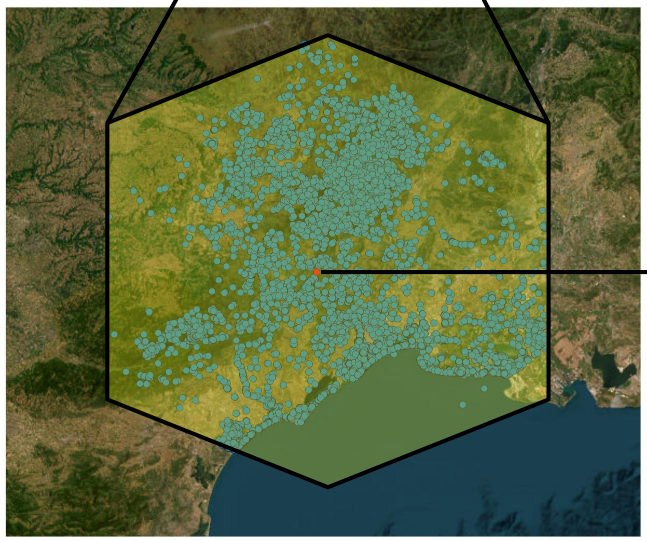

Species name	Cover %
Artemisia campestris	2.0
Asparagus officinalis	2.0
Bellardia trixago	2.0
Brachypodium phoenicoides	3.0
Dactylis glomerata	13.0
Erigeron sumatrensis	2.0
Lagurus ovatus	3.0
Medicago minima	3.0
Phillyrea angustifolia	88.0
Phragmites australis	2.0
Rostraria cristata	2.0
Silene italica	2.0

Figure 16. Hexagonal binning showing the distribution of vegetation plots in Europe and adjacent areas from the curated dataset (top). Close-up view of the raw spatial distribution of vegetation plots on a specific bin from the area around Montpellier, France (bottom-left). Further breakdown on a vegetation plot (assigned to the habitat type *S51*, i.e., *Mediterranean maquis and arborescent matorral*) with the list of co-occurring species (bottom-right). As this sample contains a ground truth label, it is used during both the fill-mask task and the text classification task (nine times in training and once in validation). Once species are ranked in descending order of cover abundance, the vegetation plot results in the following sentence: "*phillyrea angustifolia*, *dactylis glomerata*, *brachypodium phoenicoides*, *lagurus ovatus*, *medicago minima*, *artemisia campestris*, *asparagus officinalis*, *bellardia trixago*, *erigeron sumatrensis*, *phragmites australis*, *rostraria cristata*, *silene italica*".

S33 - Comparison

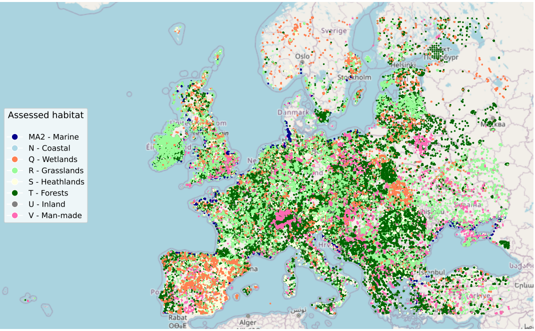

(a) Mapping of all labeled vegetation plots (i.e., the text classification dataset). The original habitat types labels are used, which were directly generated by the database coordinators using the expert system EUNIS-ESy.

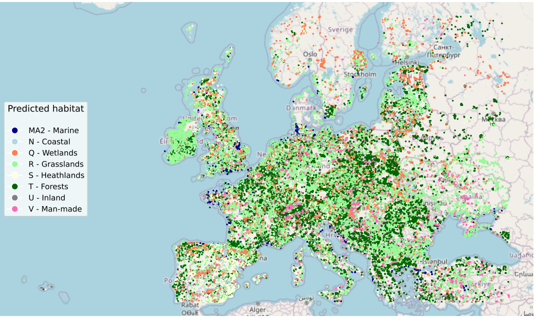

(b) Mapping of all unlabeled vegetation plots (i.e., the fill-mask dataset). The predictions output by Pl@ntBERT (large-species version trained on folds 1-9) are used.

Figure 17. Comparison of the real distribution of the vegetation plots from the labeled dataset with the predicted distribution of the vegetation plots from the unlabeled dataset for the eight broad habitat groups.

27/31

S34 - Mapping

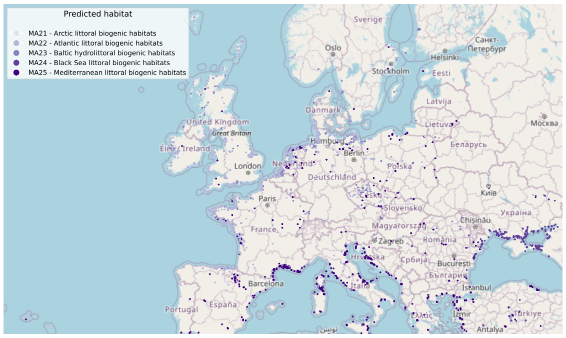

(a) Littoral habitats (MA2)

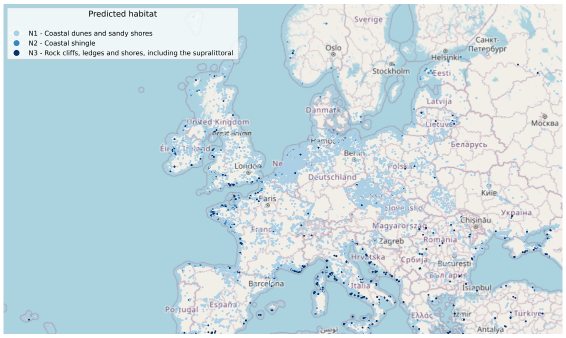

(b) Coastal habitats (N)

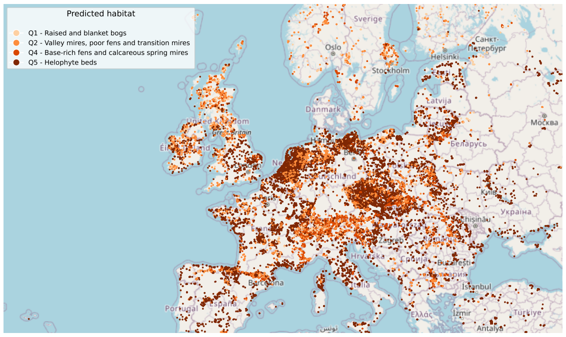

(c) Wetlands (Q)

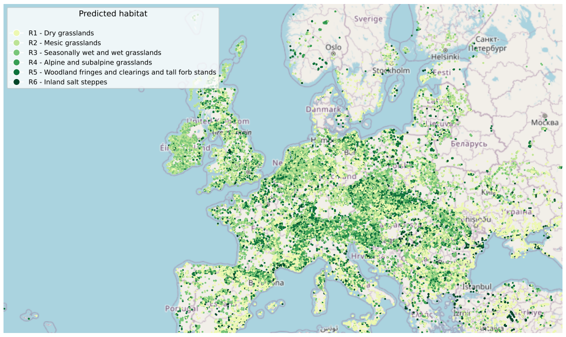

(d) Grasslands (R)

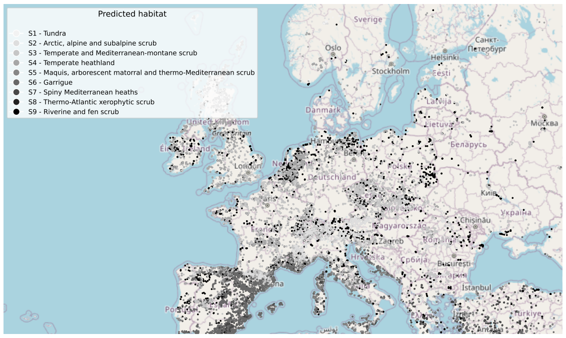

(e) Heathlands (S)

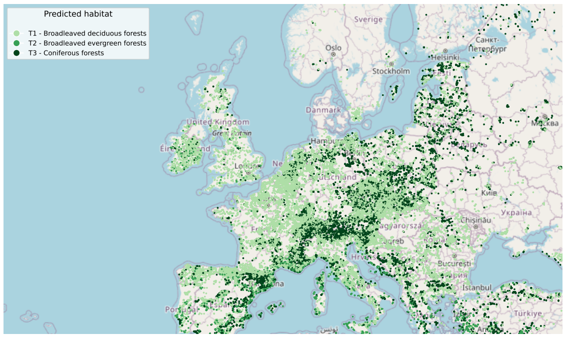

(f) Forests (T)

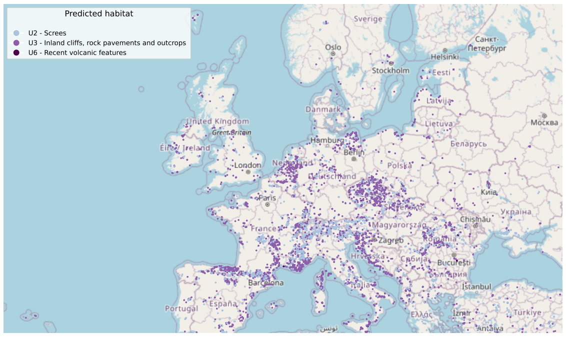

(g) Inland habitats (U)

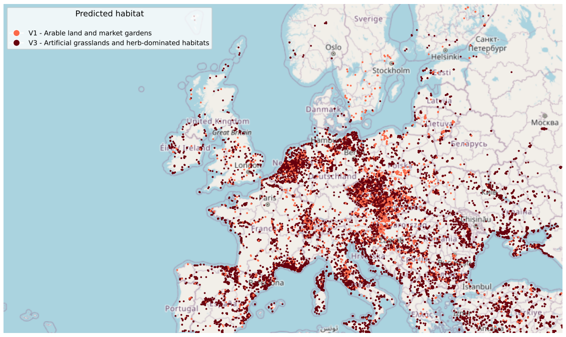

(h) Man-made habitats (V)

Figure 18. Mapping of each broad habitat group (further divided by habitat groups) by using the Pl@ntBERT model (large-species version trained on folds 1-9) to classify each vegetation plot from the fill-mask dataset.

S35 - Databases

Table 10. Overview of the EVA vegetation plots from the data selection. The name of each database is the one used in the TURBOVEG 3 (TV3) program and the code is the one used in the Global Index of Vegetation-Plot Databases (GIVD) metadatabase. A custodian is a person who owns the contributed data, or an authorised representative of all contributors of a collective database (i.e., a database containing data contributed by more than one person). Each database contributed to EVA is represented by one custodian. In total, 1,731,055 vegetation plots were included in this data selection.

Begin of Table 10

Name	Code	Custodian	# of plots	% of total
AMS-VegBank	EU-IT-021	Alessandro Chiarucci	20,462	1.2%
AgriWeedClim	EU-00-035	Michael Glaser	7,385	0.4%
Albanian Vegetation Database	EU-AL-001	Michele De Sanctis	1,324	0.1%
Ammophiletea Database	EU-00-016	Corrado Marcenò	8,504	0.5%
Austria_VINCA	EU-AT-001	Wolfgang Willner	51,702	3.0%
Balkan Dry Grassland Database	EU-00-013	Kiril Vassilev	8,183	0.5%
Balkan Vegetation Database	EU-00-019	Kiril Vassilev	15,646	0.9%
Basque Country Database	EU-00-011	Idoia Biurrun	24,277	1.4%
Beech Forest DB SE Balkan	EU-00-012	Aleksander Marinšek	1,568	0.1%
Belgium-INBOVEG	EU-BE-002	Sophie Vermeersch	18,670	1.1%
Belgium-forest	EU-BE-002	Sophie Vermeersch	7,252	0.4%
Britain_nvcd	EU-GB-001	John S. Rodwell	42,834	2.5%
Bulgarian Vegetation Database	EU-BG-001	Iva Apostolova	1,957	0.1%
CBNA	∼	Sylvain Abdulhak	2,972	0.2%
CBNMed	EU-FR-006	Olivier Argagnon	18,847	1.1%
CircumMed Forest database	EU-00-026	Gianmaria Bonari	13,667	0.8%
Croatia_mix	EU-HR-001	Zvjezdana Stančić	3,095	0.2%
Croatian Vegetation Database	EU-HR-002	Željko Škvorc	14,224	0.8%
Czechia_nvd	EU-CZ-001	Milan Chytrý	113,868	6.6%
DUMIRA	EU-NL-003	Iris de Ronde	11,742	0.7%
Denmark Naturdata	EU-DK-002	Jesper Erenskjold Moeslund	239,238	13.8%
Eastern European Steppe Database	EU-00–030	Denys Vynokurov	6,132	0.4%
EcoPlant Db	EU-FR-005	Jean-Claude Gégout	5,030	0.3%
Euro-Asian tundra VDB	00-00-004	Risto Virtanen	1,132	0.1%
European Boreal Forest Database 1	EU-00-027	Anni Kanerva Jašková	6,457	0.4%
European Boreal Forest Database 2	EU-00-027	Anni Kanerva Jašková	7,472	0.4%
European Coastal Vegetation Database-A	EU-00-017	John Janssen	6,375	0.4%
European Mire VDB	EU-00-022	Tomáš Peterka	17,695	1.0%
European Weed Vegetation Database	EU-00-028	Filip Küzmič	17,435	1.0%
Forest Vegetation Database of Belarus	EU-BY-002	Ruslan Tsvirko	1,083	0.1%
France_SOPHY	EU-FR-003	Emmanuel Garbolino	154,889	8.9%
Germany Coastal VDB	EU-DE-035	Maike Isermann	7,868	0.5%
Germany Vegetweb 1	EU-DE-013	Florian Jansen	18,417	1.1%
Germany Vegetweb 2	EU-DE-013	Friedemann Goral	10,294	0.6%
Germany_gvrd	EU-DE-014	Ute Jandt	50,107	2.9%
Germany_vegmv	EU-DE-001	Florian Jansen	50,110	2.9%
GrassPlot	EU-00-003	Jürgen Dengler	6,308	0.4%
GrassVeg.DE	EU-DE-020	Ricarda Pätsch	11,621	0.7%
Gravel Bar Database_Caucasus	EU-00-025	Veronika Kalníková	104	0.0%
Gravel Bar Vegetation Database	EU-00-025	Veronika Kalníková	1,698	0.1%
Greece_forests	EU-GR-006<br>EU-GR-007	Ioannis Tsiripidis	636	0.0%
Greece_nat	EU-GR-005	Panayotis Dimopoulos	4,881	0.3%
High Mediterranean Mountains Database	EU-00-029	Gianpietro Giusso del Galdo	946	0.1%

Continuation of Table 10				
Name	Code	Custodian	# of plots	% of total
Hungary	EU-HU-003	János Csiky	4,714	0.3%
Ireland_nvd	EU-IE-001	Úna FitzPatrick	27,085	1.6%
Italy_HabItAlp	EU-IT-010	Laura Casella	4,080	0.2%
Italy_UniRoma	EU-IT-011	Emiliano Agrillo	32,217	1.9%
Italy_mires	EU-IT-010	Laura Casella	916	0.1%
Kriti	EU-GR-001	Erwin Bergmeier	5,269	0.3%
Latvian Grassland VDB	EU-LV-001	Solvita Rūsiņa	6,738	0.4%
Lithuanian Vegetation Database	EU-LT-001	Valerius Rašomavičius	8,145	0.5%
Macedonia	EU-MK-001	Renata Ćušterevska	382	0.0%
Masaryk University Database 1	EU-00-031	Milan Chytrý	2,711	0.2%
Masaryk University Database 2	EU-00-031	Milan Chytrý	416	0.0%
MonteNegro VDB	EU-ME-001	Milica Stanišić-Vujačić	3,176	0.2%
Nenets_Tundra	AS-RU-005	Igor Lavrinenko	1,108	0.1%
Netherlands	EU-NL-001	Stephan Hennekens	147,211	8.5%
Non-forest Vegetation of Southern Poland	EU-PL-005	Remigiusz Pielech	1,927	0.1%
Nordic Vegetation Database 1	EU-00-018	Jonathan Lenoir	9,848	0.6%
Nordic Vegetation Database 2	EU-00-018	Jonathan Lenoir	2,241	0.1%
Nordic_Baltic EDGG	EU-00-002	Jürgen Dengler	10,768	0.6%
Poland	EU-PL-001	Zygmunt Kącki	77,717	4.5%
Poland Forest Database	EU-PL-003	Remigiusz Pielech	3,571	0.2%
RanVegDunes	EU-IT-020	Alicia Acosta	2,120	0.1%
Romania Grassland Database	EU-RO-008	Eszter Ruprecht	31,654	1.8%
Romania forest	EU-RO-007	Adrian Indreica	9,070	0.5%
Russia Ural nonforest	00-RU-006	Sergey Yamalov	3,259	0.2%
Russia Volga	EU-RU-002	Valentin Golub	12,927	0.7%
Russia_temperate_forests	EU-RU-014	Larisa Khanina	6,281	0.4%
SE Europe Forest DB	EU-00-021	Andraž Čarni	3,699	0.2%
SIVIM	EU-00-004	Xavier Font	3,262	0.2%
SIVIM - Alpine	EU-00-004	Borja Jiménez-Alfaro	6,021	0.3%
SIVIM - Catalonia	EU-00-004	Xavier Font	3,875	0.2%
SIVIM - Deciduous Forests	EU-00-023	Juan Antonio Campos	6,311	0.4%
SIVIM - Floodplain Forests	EU-00-024	Idoia Biurrun	3,993	0.2%
SIVIM - Grasslands	EU-ES-002	Maria Pilar Rodríguez-Rojo	12,188	0.7%
SIVIM - Macaronesia	EU-00-004	Borja Jiménez-Alfaro	3,515	0.2%
SIVIM - Sclerophyllous vegetation	EU-00-004	Federico Fernández-González	12,924	0.7%
SIVIM - Shrublands	EU-00-004	Rosario G Gavilán	10,706	0.6%
SIVIM - Wetlands	EU-ES-001	Aaron Pérez-Haase	6,663	0.4%
Schleswig-Holstein Db	EU-DE-040	Joachim Schrautzer	2,544	0.1%
Scottish Coastal Survey	EU-GB-005	Robin Pakeman	6,396	0.4%
Scottish Vegetation Resurvey	EU-GB-006	Ruth Mitchell	7,418	0.4%
Serbia_grasslands	EU-RS-002	Svetlana Aćić	5,587	0.3%
Serbian VDB	EU-RS-003<br>EU-RS-004	Mirjana Krstivojević Ćuk	1,131	0.1%
Serra da Estrella database	EU-PT-001	Jan Jansen	1,578	0.1%
Slovakia_nvd	EU-SK-001	Milan Valachovič	39,016	2.3%
Slovenia	EU-SI-001	Urban Šilc	19,930	1.2%
Steppe vegetation Rostov Region Database	∼	Olga Demina	1,145	0.1%
Switzerland Grassland DB	EU-CH-011	Ariel Bergamini	8,658	0.5%
Switzerland_forests	EU-CH-005	Thomas Wohlgemuth	14,193	0.8%
Tatarstan Vegetation Database	EU-RU-011	Vadim Prokhorov	5,640	0.3%
Teberda - Caucasus Database	∼	Vladimir Onipchenko	1,206	0.1%
Transcaucasian Vegetation Database	AS-00-005	Pavel Novák	1,944	0.1%

Continuation of Table 10				
Name	Code	Custodian	# of plots	% of total
Turkey Forest Database	00-TR-001	Ali Kavgacı	8,444	0.5%
Turkey NFVDT	00-TR-003	Behlül Güler	25,032	1.4%
Turkey Oak-Forest Database	AS-TR-002	Emin Uğurlu	1,181	0.1%
UK Floodplain Meadows Database	EU-GB-004	Irina Tatarenko	25,515	1.5%
Ukraine Grassland Database A	EU-UA-001	Anna Kuzemko	8,650	0.5%
Ukraine Grassland Database B	EU-UA-001	Anna Kuzemko	443	0.0%
Ukraine Halophytic Coastal VDB	EU-UA-005	Tetiana Dziuba	5,471	0.3%
Ukraine Psammophytic VDB	∼	Tetiana Dziuba	1,503	0.1%
Ukraine_onyshchenko	EU-UA-006	Viktor Onyshchenko	3,873	0.2%
Ukrainian Anthropogenic VDB	∼	Tetiana Dziuba	8,835	0.5%
Ukrainian Wetland Database	∼	Svitlana Yemelianova	180	0.0%
VegFrance	EU-FR-004	Jan-Bernard Bouzillé	2,839	0.2%
VegItaly	EU-IT-001	Roberto Venanzoni	11,905	0.7%
VegetWeb Germany – Tüxen´s archive	EU-DE-013	Friedemann Goral	2,683	0.2%
Viola db	EU-IT-019	Angela Stanisci	1,319	0.1%
WetVegEurope Database	EU-00-020	Flavia Landucci	3,981	0.2%
End of Table 10				

S36 - Rank

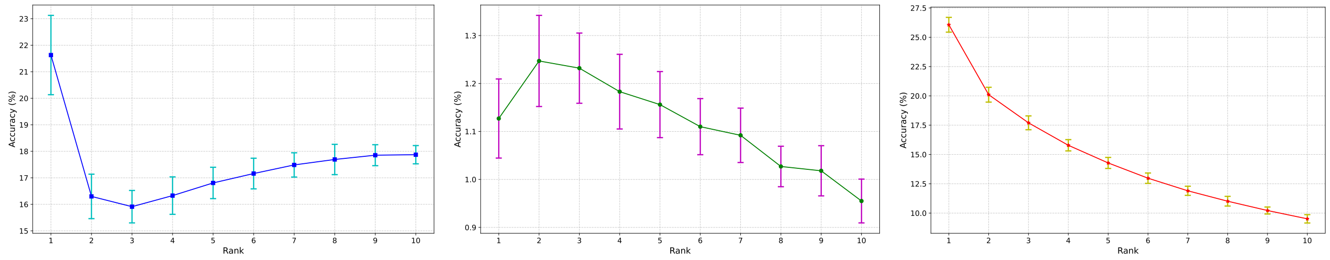

(a) Focus on the Pl@ntBERT model **(b)** Focus on the co-occurrence matrix **(c)** Focus on the neural network

Figure 19. Specific focus of the results obtained by the large-species model (19a), the co-occurrence matrix (19b), and the neural network (19c) on the masking accuracy per rank (micro-averaged over the ten cross-validation folds). Note the difference in y axis in the three graphs.

S37 - Assistance

To write the manuscript and code the framework, the authors were helped by AI tools (always under human supervision):

- **ChatGPT (OpenAI)** was employed to assist with writing tasks, such as rephrasing paragraphs for clarity, suggesting structure for sections, and drafting initial versions of standard text (e.g., method descriptions, figure legends, or introductory framing). The authors carefully reviewed and revised all AI-assisted text to ensure scientific accuracy, originality, and alignment with the study's aims. No AI tool was used to generate novel scientific hypotheses, interpret results, or make substantive contributions to the study's intellectual content.
- **Copilot (GitHub)** was used during code development to autocomplete standard functions, suggest syntax corrections, and generate boilerplate code for tasks such as data loading, training loops, and metrics computation. All code generated using Copilot was carefully reviewed, tested, and modified by the authors to ensure correctness and appropriateness for the specific research goals.

The use of these tools was limited to increasing efficiency and did not replace human expertise or critical decision-making. No AI tools were listed as authors, and their role was supportive in nature. This disclosure aligns with most guidelines for the ethical and transparent use of generative AI in scientific writing and research workflows.
