# Supplementary material for: Learning the syntax of plant assemblages
Source: Nat Plants. 2025 Oct 13;11(10):2026–40. doi: 10.1038/s41477-025-02105-7 (PMC12537494; doi:10.1038/s41477-025-02105-7)
Supplement: Supplementary file 2 — Reporting Summary [file 41477_2025_2105_MOESM2_ESM.pdf]

Corresponding author(s): César LEBLANC

Last updated by author(s): Jul 26, 2025

Reporting Summary

Nature Portfolio wishes to improve the reproducibility of the work that we publish. This form provides structure for consistency and transparency in reporting. For further information on Nature Portfolio policies, see our Editorial Policies and the Editorial Policy Checklist.

Statistics

For all statistical analyses, confirm that the following items are present in the figure legend, table legend, main text, or Methods section.

n/a	Confirmed	
☒	☐	The exact sample size (n) for each experimental group/condition, given as a discrete number and unit of measurement
☒	☐	A statement on whether measurements were taken from distinct samples or whether the same sample was measured repeatedly
☒	☐	The statistical test(s) used AND whether they are one- or two-sided<br>*Only common tests should be described solely by name; describe more complex techniques in the Methods section.*
☒	☐	A description of all covariates tested
☒	☐	A description of any assumptions or corrections, such as tests of normality and adjustment for multiple comparisons
☒	☐	A full description of the statistical parameters including central tendency (e.g. means) or other basic estimates (e.g. regression coefficient) AND variation (e.g. standard deviation) or associated estimates of uncertainty (e.g. confidence intervals)
☒	☐	For null hypothesis testing, the test statistic (e.g. F, t, r) with confidence intervals, effect sizes, degrees of freedom and P value noted<br>*Give P values as exact values whenever suitable.*
☒	☐	For Bayesian analysis, information on the choice of priors and Markov chain Monte Carlo settings
☒	☐	For hierarchical and complex designs, identification of the appropriate level for tests and full reporting of outcomes
☒	☐	Estimates of effect sizes (e.g. Cohen's d, Pearson's r), indicating how they were calculated

Our web collection on statistics for biologists contains articles on many of the points above.

Software and code

Policy information about availability of computer code

Data collection	No software was used for data collection.
Data analysis	Pl@ntBERT (our own software created for this study available here: https://github.com/cesar-leblanc/PlantBERT) was used for data analysis.

For manuscripts utilizing custom algorithms or software that are central to the research but not yet described in published literature, software must be made available to editors and reviewers. We strongly encourage code deposition in a community repository (e.g. GitHub). See the Nature Portfolio guidelines for submitting code & software for further information.

Data

Policy information about availability of data

All manuscripts must include a data availability statement. This statement should provide the following information, where applicable:

- Accession codes, unique identifiers, or web links for publicly available datasets
- A description of any restrictions on data availability
- For clinical datasets or third party data, please ensure that the statement adheres to our policy

The data that support the findings of this study are available from EVA but restrictions apply to the availability of these data, which were used under license for the current study, and so are not publicly available. Data are, however, available from the authors or EVA custodians upon reasonable request and with permission of EVA. The DOI of the EVA data selection for this project is https://doi.org/10.58060/QR4B-G979.

Research involving human participants, their data, or biological material

Policy information about studies with human participants or human data. See also policy information about sex, gender (identity/presentation), and sexual orientation and race, ethnicity and racism.

Reporting on sex and gender	This field doesn't apply to our study, as its focus was plants.
Reporting on race, ethnicity, or other socially relevant groupings	This field doesn't apply to our study, as its focus was plants.
Population characteristics	This field doesn't apply to our study, as its focus was plants.
Recruitment	This field doesn't apply to our study, as its focus was plants.
Ethics oversight	This field doesn't apply to our study, as its focus was plants.

Note that full information on the approval of the study protocol must also be provided in the manuscript.

Field-specific reporting

Please select the one below that is the best fit for your research. If you are not sure, read the appropriate sections before making your selection.

☐ Life sciences ☐ Behavioural & social sciences ☒ Ecological, evolutionary & environmental sciences

For a reference copy of the document with all sections, see nature.com/documents/nr-reporting-summary-flat.pdf

Ecological, evolutionary & environmental sciences study design

All studies must disclose on these points even when the disclosure is negative.

Study description	We studied the communities and habitat types (EUNIS typology) of 1,423,164 vegetation plots (covering 29,149,022 observations of 14,189 different plant species). There were two different goals:<br>- identifying likely missing species of the vegetation plots and<br>- identifying the habitat type of the vegetation plots.
Research sample	This study covers most of European flora. The biggest study group (i.e., phylum) was Tracheophyta, with 12,546 different species. The dataset represents pretty well the vegetation of Europe.
Sampling strategy	The data comes from the European Vegetation Archive, where different networks of experts did in-situ vegetation sampling.
Data collection	Vegetation-plot data for this study were provided by Sylvain Abdulhak, Alicia Acosta, Emiliano Agrillo, Pierangela Angelini, Iva Apostolova, Olivier Argagnon, Fabio Attorre, Svetlana Aćić, Christian Berg, Ariel Bergamini, Erwin Bergmeier, Idoia Biurrun, Maxim Bobrovsky, Steffen Boch, Gianmaria Bonari, Anne Bonis, Zoltán Botta-Dukát, Jan-Bernard Bouzillé, Helge Bruelheide, Vanessa Bruzzaniti, Juan Antonio Campos, Andraž Čarni, Maria Laura Carranza, Laura Casella, Alessandro Chiarucci, Andrei Chuvashov, Milan Chytrý, János Csiky, Mirjana Krstivojević Ćuk, Renata Ćušterevska, Olga Demina, Jürgen Dengler, Panayotis Dimopoulos, Dmytro Dubyna, Tetiana Dziuba, Alexei Egorov, Rasmus Ejrnæs, Franz Essl, Jörg Ewald, Giuliano Fanelli, Federico Fernández-González, Úna FitzPatrick, Xavier Font, Gianpietro Giusso del Galdo, Emmanuel Garbolino, Itziar García-Mijangos, Rosario G. Gavilán, Jean-Michel Genis, Michael Glaser, Valentin Golub, Friedemann Goral, Jean-Claude Gégout, Behlül Güler, Rense Haveman, Stephan Hennekens, Adrian Indreica, Maike Isermann, Ute Jandt, Florian Jansen, Jan Jansen, John Janssen, Anni Kanerva Jašková, Borja Jiménez-Alfaro, Martin Jiroušek, Veronika Kalníková, Ali Kavgacı, Larisa Khanina, Ilona Knollová, Vitaliy Kolomiychuk, Łukasz Kozub, Daniel Krstonošić, Helmut Kudrnovsky, Anna Kuzemko, Filip Küzmič, Zygmunt Kącki, Flavia Landucci, Igor Lavrinenko, Mariya Lebedeva, Jonathan Lenoir, Armin Macanović, Corrado Marcenò, Aleksander Marinšek, Marco Massimi, Ruth Mitchell, Jesper Erenskjold Moeslund, Pavel Novák, Vladimir Onipchenko, Viktor Onyshchenko, Robin Pakeman, Hristo Pedashenko, Tomáš Peterka, Remigiusz Pielech, Vadim Prokhorov, Ricarda Pätsch, Aaron Pérez-Haase, Valerijus Rašomavičius, Maria Pilar Rodríguez-Rojo, John S. Rodwell, Iris de Ronde, Eszter Ruprecht, Solvita Rūsiņa, Michele De Sanctis, Joop Schaminée, Joachim Schrautzer, Ingrid Seynave, Jozef Šibík, Urban Šilc, Željko Škvorc, Desislava Sopotlieva, Angela Stanisci, Milica Stanišić-Vujačić, Zvjezdana Stančić, Zora Dajić Stevanović, Danijela Stešević, Jens-Christian Svenning, Grzegorz Swacha, Irina Tatarenko, Ioannis Tsiripidis, Ruslan Tsvirko, Pavel Dan Turtureanu, Domas Uogintas, Emin Uğurlu, Milan Valachovič, Kiril Vassilev, Roberto Venanzoni, Sophie Vermeersch, Risto Virtanen, Denys Vynokurov, Lynda Weekes, Wolfgang Willner, Thomas Wohlgemuth, Sergey Yamalov, Svitlana Yemelianova, and Dominik Zukal. The cover of individual species was, in most vegetation plots, recorded using a cover-abundance scale (in most cases, using the Braun-Blanquet scale). The vegetation plots are usually between 10 and 400 square meters.
Timing and spatial scale	Vegetation plots used in this study were collected between 1873 and 2022 in Europe and adjacent countries.
Data exclusions	The data comes from the European Vegetation Archive. Vegetation plots outside of Europe and adjacent areas were excluded. Vegetation plots which were not georeferenced were excluded. Species with a given cover percentage of 0 were excluded, assuming these were errors or scientists reporting absent species. Species which we could not harmonize using the GBIF Backbone Taxonomy were excluded. Taxa identified only to the genus level were dropped, and taxa identified at the subspecies level were lumped

together at the species level (e.g., Hedera was dropped but both Hedera helix subsp. helix and Hedera helix subsp. poetarum were merged into Hedera helix). Hybrid species and very rare species (i.e., species that appeared less than ten times in the whole dataset) were excluded. Vegetation plots that lost more than 25% of their taxa or their most abundant taxon after the species names matching were removed from the dataset to ensure that the remaining plots still provided reliable representations of vegetation patterns.

Reproducibility

The used seeds for Python, NumPy, and PyTorch are provided in the source code (as the default seeds). The DOI of the dataset is provided in the paper. Running the code with these seeds and with the same data extraction will result in the same experimental findings.

Randomization

The set of labeled vegetation plots was strategically split. As the quantity of available full lists of plant species with estimates of cover-abundance of each species and habitat type assignment is not very high (i.e., less than 1M vegetation plots for all of Europe, a relatively low number compared to the vast amount of biodiversity data available), partitioning the available data into a training set and a test set would reduce the number of training samples to a level too low for effective model training. As a result, we instead used k-fold cross-validation (CV) to split the dataset into 10 subsets. Then, for each of the splits, the models were trained using 9 of the subsets for training and the latter one for validation. However, cross-validation scores for the classification of vegetation plots are biased if the data is randomly split, because they are commonly spatially autocorrelated (spatially closer data points have similar values). To reduce the bias, we split data along spatial blocks. This procedure avoids fitting structural patterns and allows the separation of near-duplicates. Such vegetation plots differ from each other in a very small portion of species (e.g., if they are close in space, two vegetation plots may exhibit identical plant composition but feature species with slightly contrasting abundances). The data set was thus first split into spatial blocks of 6 arc-minutes (0.1 degree on the World Geodetic System 1984, or WGS 84, spheroid). Then, the blocks were split into folds. Since the geographic distribution of vegetation plots across Europe is unequal, each block can have a different number of data points. The folds were thus balanced to have approximately equal number of plots instead of assigning the same number of blocks to each fold (which could have led to folds with very different numbers of data points).

Blinding

During the first fine-tuning phase of our workflow, we trained the models by following a fill-mask task. In each vegetation plot, we masked 15% of the tokens (except for commas, the classify tokens [CLS], which represent entire input sequences, and the separate tokens [SEP], which mark the separation between different input sequences). These masked tokens consisted of full species names in the case of Pl@ntBERT-species and of genus names or species epithets in the case of Pl@ntBERT-term. We followed the same procedure used in the original BERT paper: each selected token was replaced by (i) the [MASK] token 80% of the time, (ii) a random species in the case of Pl@ntBERT-species or a random genus name or species epithet in the case of Pl@ntBERT-term 10% of the time, or (iii) the same species 10% of the time.

Did the study involve field work? ☐ Yes ☒ No

Reporting for specific materials, systems and methods

We require information from authors about some types of materials, experimental systems and methods used in many studies. Here, indicate whether each material, system or method listed is relevant to your study. If you are not sure if a list item applies to your research, read the appropriate section before selecting a response.

Materials & experimental systems

n/a	Involved in the study
☒	☐ Antibodies
☒	☐ Eukaryotic cell lines
☒	☐ Palaeontology and archaeology
☒	☐ Animals and other organisms
☒	☐ Clinical data
☒	☐ Dual use research of concern
☒	☐ Plants

Methods

n/a	Involved in the study
☒	☐ ChIP-seq
☒	☐ Flow cytometry
☒	☐ MRI-based neuroimaging

Plants

Seed stocks

This field doesn't apply to our study as we didn't use any seed stock.

Novel plant genotypes

This field doesn't apply to our study as we didn't produce any novel plant genotype.

Authentication

This field doesn't apply to our study as we didn't generate any seed stock or novel plant genotype.
